# Supplementary material for: Unveiling a Microexon Switch: Novel Regulation of the Activities of Sugar Assimilation and Plant-Cell-Wall-Degrading Xylanases and Cellulases by Xlr2 in Trichoderma virens
Source: Int J Mol Sci. 2024 May 9;25(10):5172. doi: 10.3390/ijms25105172 (PMC11121469; doi:10.3390/ijms25105172)
Supplement: Supplementary file 1 [file ijms-25-05172-s001.zip › Figure S1 new number.pdf]

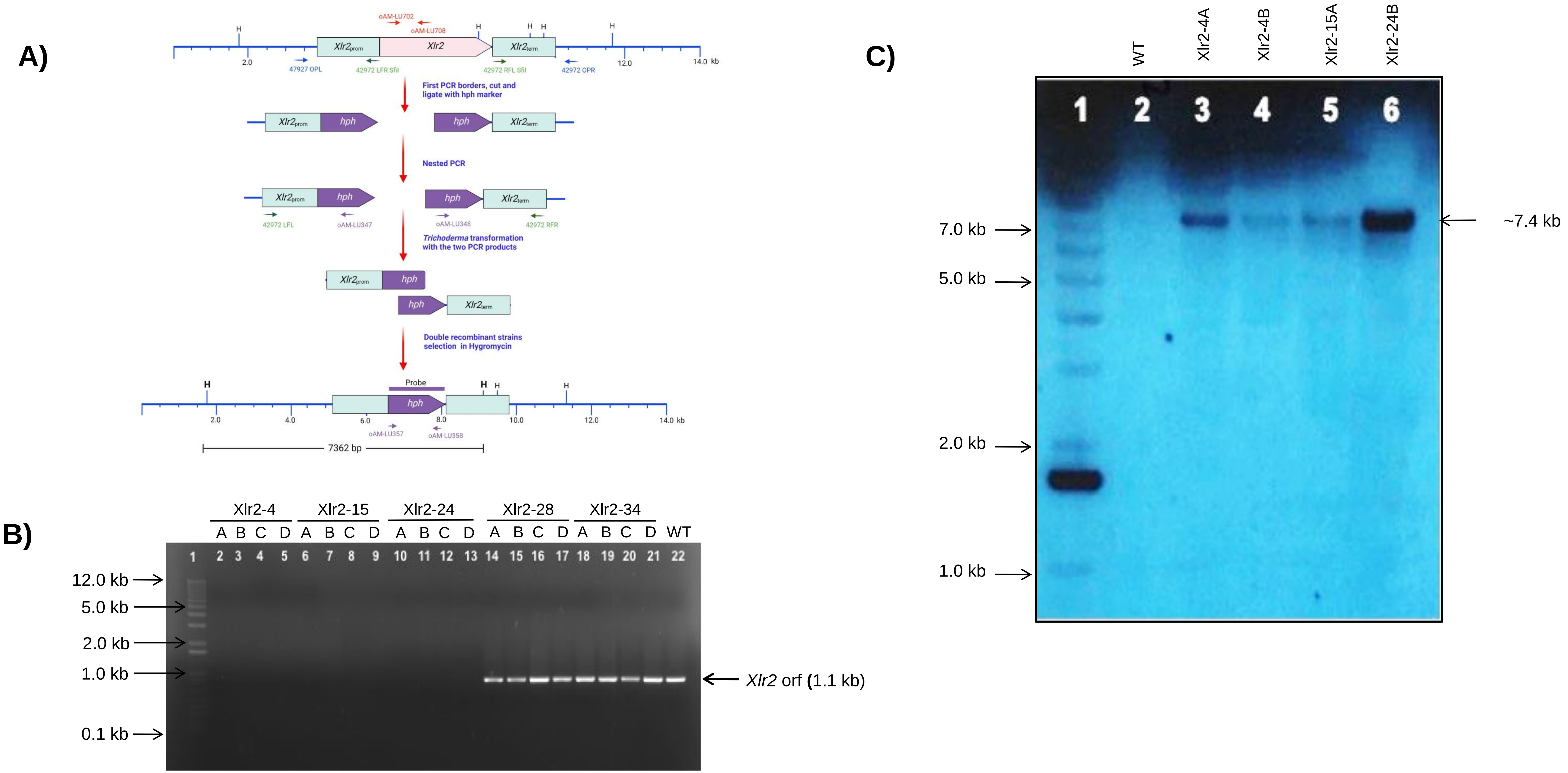

Figure S1. *Trichoderma virens* Gv29.8 Xlr2 deletion mutants and selection strategy. A) Diagram of integration events and primer locations. The colours of the primers represent the DNA templates used. **B)** Amplification of *Xlr2* ORF using the oAM-LU702/oAM-LU708 primer mixture and genomic DNA from single isolated strains regenerated on PDA supplemented with 100 µg/ml of hygromycin. Four to six rounds of single spore isolation were performed before DNA isolation. The absence of Xlr2 was observed in lanes 2 to 13, corresponding to three strains sub-isolated from three individual recombination events. Genomic DNA was used to verify the absence of the Xlr2 gene. Lane 1: 1 Kb Plus DNA ladder (Invitrogen), Lanes 2-21: candidate *Xlr2* deletion mutant strains, Lane 22: Wild Type (WT) genomic DNA. **C)** A 7.36 kb band, as indicated in A, was identified by Southern blotting, using Hind*III*-digested genomic DNA from the indicated lines. The Southern blot was probed using an *hph* probe (generated with the primers oAM-LU357 and oAM-LU358) and labelled using the PCR DIG labelling mix system (Roche). Lane 1: Molecular Weight Marker, Lane 2: wild strain, Lane 3: ΔXlr2-4A, Lane 4: ΔXlr2-4B, Lane 5: ΔXlr2-15A, Lane 6: ΔXlr2-24B.
