# Supplementary material for: Unveiling a Microexon Switch: Novel Regulation of the Activities of Sugar Assimilation and Plant-Cell-Wall-Degrading Xylanases and Cellulases by Xlr2 in Trichoderma virens
Source: Int J Mol Sci. 2024 May 9;25(10):5172. doi: 10.3390/ijms25105172 (PMC11121469; doi:10.3390/ijms25105172)
Supplement: Supplementary file 1 [file ijms-25-05172-s001.zip › Figure S2 new number.pdf]

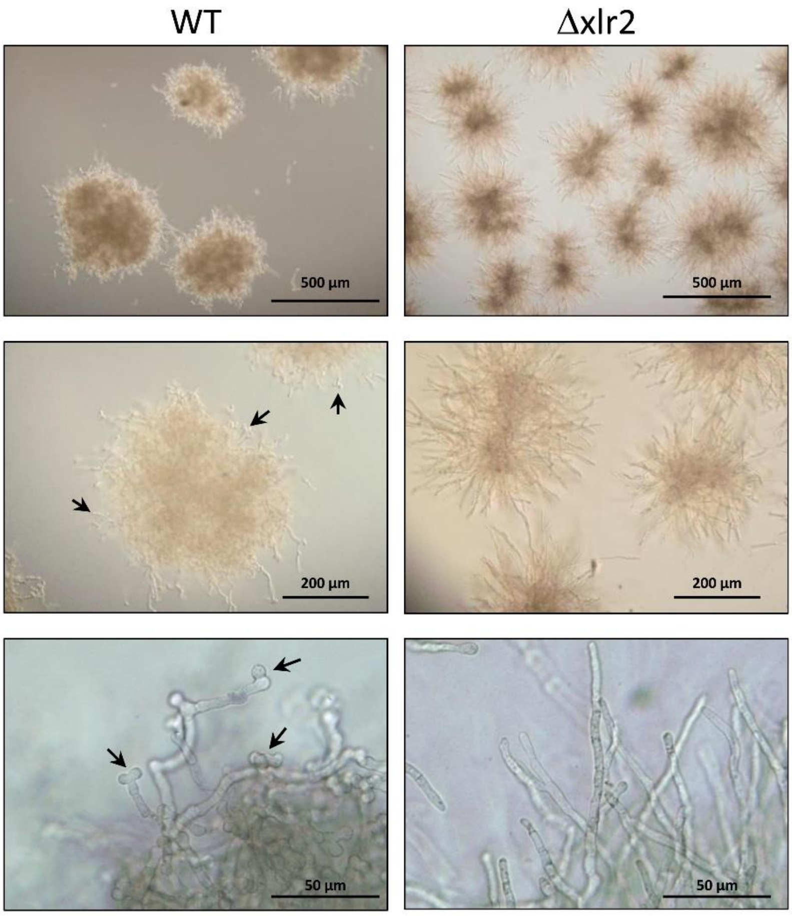

Figure S2. Submerged growth of the WT (wild-type) and Δxlr2 strains of *Trichoderma virens* Gv29.8. The strains were cultured in liquid media for 72 hours in Potato Dextrose Broth at 25 °C with agitation at 150 rpm. Images were captured using an Olympus BX51 Fluorescence Microscope. Arrows indicate the tips of the WT strain, displaying the characteristic swelling typical of the Gv29.8 strain.
