# Supplementary material for: Unveiling a Microexon Switch: Novel Regulation of the Activities of Sugar Assimilation and Plant-Cell-Wall-Degrading Xylanases and Cellulases by Xlr2 in Trichoderma virens
Source: Int J Mol Sci. 2024 May 9;25(10):5172. doi: 10.3390/ijms25105172 (PMC11121469; doi:10.3390/ijms25105172)
Supplement: Supplementary file 1 [file ijms-25-05172-s001.zip › Figure S3 new number.pdf]

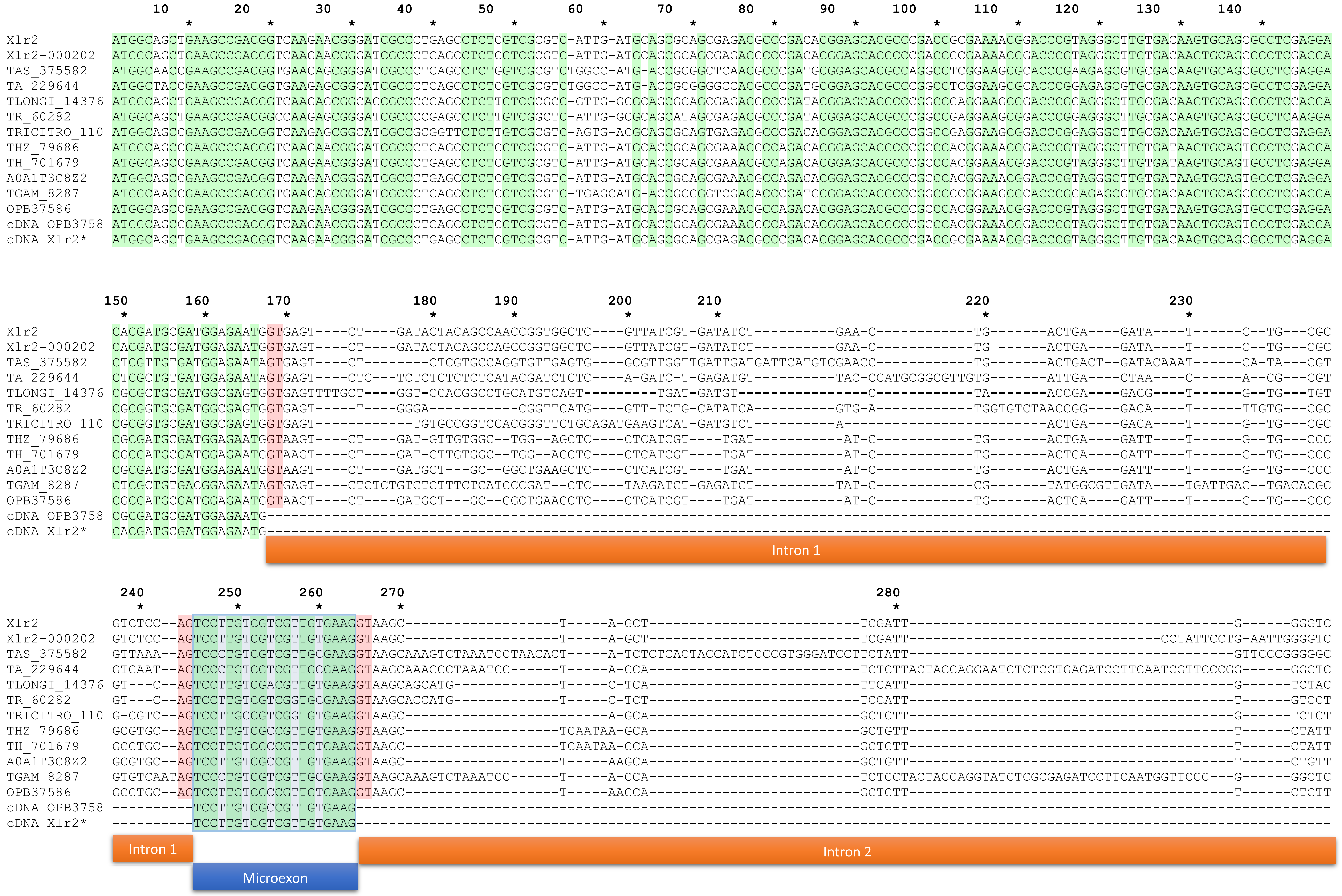

10 20 30 40 50 60 70 80 90 100 110 120 130 140
Xlr2 ATGGCAGCTGAAGCCGACGGTCAAGAACGGGATCGCCCTGAGCCTCTCGTCGCGTC-ATTG-ATGCAGCGCAGCGAGACGCCCGACACGGAGCACGCCCGACCGCGAAAACGGACCCGTAGGGCTTGTGACAAGTGCAGCGCCTCGAGGA
Xlr2-000202 ATGGCAGCTGAAGCCGACGGTCAAGAACGGGATCGCCCTGAGCCTCTCGTCGCGTC-ATTG-ATGCAGCGCAGCGAGACGCCCGACACGGAGCACGCCCGACCGCGAAAACGGACCCGTAGGGCTTGTGACAAGTGCAGCGCCTCGAGGA
TAS_375582 ATGGCAACCGAAGCCGACGGTGAACAGCGGGATCGCCCTCAGCCTCTGGTCGCGTCTGGCC-ATG-ACCGCGGCTCAACGCCCGATGCGGAGCACGCCAGGCCTCGGAAGCGCACCCGAAGAGCGTGCGACAAGTGCAGCGCCTCGAGGA
TA_229644 ATGGCTACCGAAGCCGACGGTGAAGAGCGGCATCGCCCTCAGCCTCTCGTCGCGTCTGGCC-ATG-ACCGCGGGGCCACGCCCGATGCGGAGCACGCCCGGCCTCGGAAGCGCACCCGGAGAGCGTGCGACAAGTGCAGCGCCTCGAGGA
TLONGI_14376 ATGGCAGCTGAAGCCGACGGTCAAGAGCGGCACCGCCCCGAGCCTCTTGTCGCGCC-GTTG-GCGCAGCGCAGCGAGACGCCCGATACGGAGCACGCCCGGCCGAGGAAGCGGACCCGGAGGGCTTGCGACAAGTGCAGCGCCTCCAGGA
TR_60282 ATGGCAGCTGAAGCCGACGGCCAAGAGCGGGATCGCCCCGAGCCTCTTGTCGGCTC-ATTG-GCGCAGCATAGCGAGACGCCCGATACGGAGCACGCCCGGCCGAGGAAGCGGACCCGGAGGGCTTGCGACAAGTGCAGCGCCTCAAGGA
TRICITRO_110 ATGGCAGCCGAAGCCGACGGTCAAGAGCGGCATCGCCCGCGGTTCTCTTGTCGCGTC-AGTG-ACGCAGCGCAGTGAGACGCCCGACACGGAGCACGCCCGGCCGAGGAAGCGGACCCGGAGGGCTTGCGACAAGTGCAGCGCCTCGAGGA
THZ_79686 ATGGCAGCCGAAGCCGACGGTCAAGAACGGGATCGCCCTGAGCCTCTCGTCGCGTC-ATTG-ATGCACCGCAGCGAAACGCCAGACACGGAGCACGCCCGCCCACGGAAACGGACCCGTAGGGCTTGTGATAAGTGCAGTGCCTCGAGGA
TH_701679 ATGGCAGCCGAAGCCGACGGTCAAGAACGGGATCGCCCTGAGCCTCTCGTCGCGTC-ATTG-ATGCACCGCAGCGAAACGCCAGACACGGAGCACGCCCGCCCACGGAAACGGACCCGTAGGGCTTGTGATAAGTGCAGTGCCTCGAGGA
A0A1T3C8Z2 ATGGCAGCCGAAGCCGACGGTCAAGAACGGGATCGCCCTGAGCCTCTCGTCGCGTC-ATTG-ATGCACCGCAGCGAAACGCCAGACACGGAGCACGCCCGCCCACGGAAACGGACCCGTAGGGCTTGTGATAAGTGCAGTGCCTCGAGGA
TGAM_8287 ATGGCAACCGAAGCCGACGGTGAACAGCGGGATCGCCCTCAGCCTCTCGTCGCGTC-TGAGCATG-ACCGCGGGTCGACACCCGATGCGGAGCACGCCCGGCCCGGAAGCGCACCCGGAGAGCGTGCGACAAGTGCAGCGCCTCGAGGA
OPB37586 ATGGCAGCCGAAGCCGACGGTCAAGAACGGGATCGCCCTGAGCCTCTCGTCGCGTC-ATTG-ATGCACCGCAGCGAAACGCCAGACACGGAGCACGCCCGCCCACGGAAACGGACCCGTAGGGCTTGTGATAAGTGCAGTGCCTCGAGGA
cDNA OPB3758 ATGGCAGCCGAAGCCGACGGTCAAGAACGGGATCGCCCTGAGCCTCTCGTCGCGTC-ATTG-ATGCACCGCAGCGAAACGCCAGACACGGAGCACGCCCGCCCACGGAAACGGACCCGTAGGGCTTGTGATAAGTGCAGTGCCTCGAGGA
cDNA Xlr2* ATGGCAGCTGAAGCCGACGGTCAAGAACGGGATCGCCCTGAGCCTCTCGTCGCGTC-ATTG-ATGCAGCGCAGCGAGACGCCCGACACGGAGCACGCCCGACCGCGAAAACGGACCCGTAGGGCTTGTGACAAGTGCAGCGCCTCGAGGA
150 160 170 180 190 200 210 220 230
Xlr2 CACGATGCGATGGAGAATGGTGAGT----CT----GATACTACAGCCAACCGGTGGCTC----GTTATCGT-GATATCT----------GAA-C------------TG-------ACTGA----GATA----T------C--TG---CGC
Xlr2-000202 CACGATGCGATGGAGAATGGTGAGT----CT----GATACTACAGCCAGCCGGTGGCTC----GTTATCGT-GATATCT----------GAA-C------------TG -------ACTGA----GATA----T------C--TG---CGC
TAS_375582 CTCGTTGTGATGGAGAATAGTGAGT----CT--------CTCGTGCCAGGTGTTGAGTG----GCGTTGGTTGATTGATGATTCATGTCGAACC------------TG-------ACTGACT--GATACAAAT------CA-TA---CGT
TA_229644 CTCGCTGTGATGGAGAATAGTGAGT----CTC---TCTCTCTCTCATACGATCTCTC----A-GATC-T-GAGATGT----------TAC-CCATGCGGCGTTGTG-------ATTGA----CTAA----C------A--CG---CGT
TLONGI_14376 CGCGCTGCGATGGCGAGTGGTGAGTTTTGCT----GGT-CCACGGCCTGCATGTCAGT---------TGAT-GATGT----------------C------------TA-------ACCGA----GACG----T------G--TG---TGT
TR_60282 CGCGGTGCGATGGCGAGTGGTGAGT-----T----GGGA-----------CGGTTCATG----GTT-TCTG-CATATCA----------GTG-A------------TGGTGTCTAACCGG----GACA----T------TTGTG---CGC
TRICITRO_110 CGCGGTGCGATGGCGAGTGGTGAGT------------TGTGCCGGTCCACGGGTTCTGCAGATGAAGTCAT-GATGTCT----------A-------------------------ACTGA----GACA----T------G--TG---CGC
THZ_79686 CGCGATGCGATGGAGAATGGTAAGT----CT----GAT-GTTGTGGC--TGG--AGCTC----CTCATCGT----TGAT----------AT-C------------TG-------ACTGA----GATT----T------G--TG---CCC
TH_701679 CGCGATGCGATGGAGAATGGTAAGT----CT----GAT-GTTGTGGC--TGG--AGCTC----CTCATCGT----TGAT----------AT-C------------TG-------ACTGA----GATT----T------G--TG---CCC
A0A1T3C8Z2 CGCGATGCGATGGAGAATGGTAAGT----CT----GATGCT---GC--GGCTGAAGCTC----CTCATCGT----TGAT----------AT-C------------TG-------ACTGA----GATT----T------G--TG---CCC
TGAM_8287 CTCGCTGTGACGGAGAATAGTGAGT----CTCTCTGTCTCTTTCTCATCCCGAT--CTC----TAAGATCT-GAGATCT----------TAT-C------------CG-------TATGGCGTTGATA----TGATTGAC--TGACACGC
OPB37586 CGCGATGCGATGGAGAATGGTAAGT----CT----GATGCT---GC--GGCTGAAGCTC----CTCATCGT----TGAT----------AT-C------------TG-------ACTGA----GATT----T------G--TG---CCC
cDNA OPB3758 CGCGATGCGATGGAGAATG---
cDNA Xlr2* CACGATGCGATGGAGAATG---
Intron 1
240 250 260 270 280
Xlr2 GTCTCC--AGTCCTTGTCGTCGTTGTGAAGGTAAGC-------------------T-----A-GCT--------------------------TCGATT--G------GGGTC
Xlr2-000202 GTCTCC--AGTCCTTGTCGTCGTTGTGAAGGTAAGC-------------------T-----A-GCT--------------------------TCGATT-------------------------------CCTATTCCTG-AATTGGGGTC
TAS_375582 GTTAAA--AGTCCCTGTCGTCGTTGCGAAGGTAAGCAAAGTCTAAATCCTAACACT-----A-TCTCTCACTACCATCTCCCGTGGGATCCTTCTATT---GTTCCCGGGGGC
TA_229644 GTGAAT--AGTCCCTGTCGTCGTTGCGAAGGTAAGCAAAGCCTAAATCC------T-----A-CCA--------------------------TCTCTTACTACCAGGAATCTCTCGTGAGATCCTTCAATCGTTCCCGG------GGCTC
TLONGI_14376 GT---C--AGTCCTTGTCGACGTTGTGAAGGTAAGCAGCATG-------------T-----C-TCA--------------------------TTCATT--G------TCTAC
TR_60282 GT---C--AGTCCTTGTCGTCGGTGCGAAGGTAAGCACCATG-------------T-----C-TCT--------------------------TCCATT--T------GTCCT
TRICITRO_110 G-CGTC--AGTCCTTGCCGTCGGTGTGAAGGTAAGC-------------------------A-GCA--------------------------GCTCTT--G------TCTCT
THZ_79686 GCGTGC--AGTCCTTGTCGCCGTTGTGAAGGTAAGC-------------------TCAATAA-GCA--------------------------GCTGTT--T------CTATT
TH_701679 GCGTGC--AGTCCTTGTCGCCGTTGTGAAGGTAAGC-------------------TCAATAA-GCA--------------------------GCTGTT--T------CTATT
A0A1T3C8Z2 GCGTGC--AGTCCTTGTCGCCGTTGTGAAGGTAAGC-------------------T-----AAGCA--------------------------GCTGTT--T------CTGTT
TGAM_8287 GTGTCAATAGTCCCTGTCGTCGTTGCGAAGGTAAGCAAAGTCTAAATCC------T-----A-CCA--------------------------TCTCCTACTACCAGGTATCTCGCGAGATCCTTCAATGGTTCCC---G------GGCTC
OPB37586 GCGTGC--AGTCCTTGTCGCCGTTGTGAAGGTAAGC-------------------T-----AAGCA--------------------------GCTGTT--T------CTGTT
cDNA OPB3758 ----------TCCTTGTCGCCGTTGTGAAG---
cDNA Xlr2* ----------TCCTTGTCGTCGTTGTGAAG---
Intron 1
Microexon
Intron 2

```
                   290                                                                                                  300                          310
                     *                                                                                                    *                            *
Xlr2         ---TC---CTGA---------------ATTGGG----------------------------------------------------------------------------GT--------CTC-CC---------T-CTC-T-GCCTCAT-
Xlr2-000202  ---TC---CCTC-------------------------------------------------------------------------------------------                               T-CT----GCCTCAT
TAS_375582   ---TC---CATT---------------CTTGGATGGAGATAGTAAC---------------------------------------------------------------GG--------CCC-CCAA-------T-CTC-C-ACCCCAT-
TA_229644    CATTC---CTGG---------------ATGGAGATAGCAACGGCCCCCAATCTCCACCTTGATGGCTCAATCCCACGTCATCTTTTCAACTGTCATATCTAACACGTTTGT--------CTT-TC---------T-CTC-T--CCTC---
TLONGI_14376 C--TC---CACA---------------ACTGGG----------------------------------------------------------------------------AT--------CTC--------------------TTCTCCT-
TR_60282     ---TCTTCCTGG---------------AATGCG----------------------------------------------------------------------------GC--------AAC-CC---------CCCTC-T--TCTCAT-
TRICITRO_110 ---TC---CATT---------------GTTCGC----------------------------------------------------------------------------TTC-------CTCTCCTG-------T-CTCATATCCTCATA
THZ_79686    ---TC---CACATTCCGGTTTCCACCCATTGGG----------------------------------------------------------------------------GC--------TTC-ATCTTCATTCAT-CTC-T-GCTTCAT-
TH_701679    ---TC---CACATTCCGGTTTCCACCCATTGGG----------------------------------------------------------------------------GC--------TTC-ATCTTCATTCAT-CTC-T-GCTTCAT-
A0A1T3C8Z2   ---TC---CACATTCTATTTTCCCCC-ATTGGA----------------------------------------------------------------------------GC--------TTC-ATCTTACTTCAT-CTC-T-GCTTCAT-
TGAM_8287    CATTC---CTGG---------------ATGGAG----------------------------------------------------------------------------ATAGCAACGGCCC-CC---------A-ATC-T-CCACCATC
OPB37586     ---TC---CACATTCTATTTTCCCCC-ATTGGA----------------------------------------------------------------------------GC--------TTC-ATCTTACTTCAT-CTC-T-GCTTCAT-
cDNA OPB3758 ------------------------------------------------------------------------------------------------------------------------------------------------------
cDNA Xlr2*   ------------------------------------------------------------------------------------------------------------------------------------------------------
```

Intron 2

```
             320                               330                          340                             350                                     360       370
               *                                 *                            *                               *                                         *         *
Xlr2         -TGATTCA-----------C-------------TTGA-AAGC--------C--TA-----T-T--GTA-----T--C-T--------AAC---T---T-C-C---C--T-----C--------G------------AGACTACGGATATA
Xlr2-000202  -TGATTCA-----------C-------------TTGA-AAGC--------C—TA------T-T—GTA------T—C—T---------AAC---T---T-C-C---C—T------C--------G------------AGACTACGGATATA
TAS_375582   -GGCTTCATCCCACGTCATC-------------TTCA-TAACTGTCATCTC--TAACACGT-T--GTG-----T--C-T--------TTC---T---T-T-C---T--T-----T--------CTCCGCTGTTTGAAGACTACGGATATA
TA_229644    ----------------------------------------------------------------------------------------TC---T---T-C-A----------------------------------AGACTACGGATACA
TLONGI_14376 -GGTTTCA-----------T-------------TCCA-CATCTCGCCC--C--TA-----T-T--ATA-----C--C-TTCTCTAACACC---T---C-G-C---C--T-----C--------CACTACCA-----AGACTACGGATACA
TR_60282     -TGTCTCA-----------CCTCA---------TACC-AATC--------A--TA-----CAT--GTACCTTCT--C-T--------AAC---A---T-G-C---T--G-----C--------CTCTGCCATCA--AGACTACGGATACA
TRICITRO_110 -TCATTCC-----------C-------------ATCATACTA--------C--TT-----TCT--CTAAC---GTGC-T--------GCC---T---C-CGCCG-C--T-----C--------A------------AGACTACGGATACA
THZ_79686    -TGTTTCA-----------CT------------TTCA-AAGCTTG-----ACGTA-----T-CTAACA-----T--CTT--------ACCCTTT---T-C-ACTACTTT-----CA-------A------------AGACTACGGATACA
TH_701679    -TGTTTCA-----------CT------------TTCA-AAGCTTG-----ACGTA-----T-CTAACA-----T--CTT--------ACCCTTT---T-C-ACTACTTT-----CA-------A------------AGACTACGGATACA
A0A1T3C8Z2   -TGTTTCA-----------CT------------TTCA-AAGC--------T--TG-----G-C--GTA-----T--C-T--------AACACAT---TAC-C---C--TTTTTACCACTTTCAA------------AGACTACGGATACA
TGAM_8287    CTGGTTTT-----------CATCCCACGTCATCTTGT-AA----------C--TG-----T-C--ATA-----T--C-T--------AAC---ACTGT-C-T---T--T-----C--------TCATCA-------AGACTACGGATACA
OPB37586     -TGTTTCA-----------CT------------TTCA-AAGC--------T--TG-----G-C--GTA-----T--C-T--------AACACAT---TAC-C---C--TTTTTACCACTTTCAA------------AGACTACGGATACA
cDNA OPB3758 ------------------------------------------------------------------------------------------------------------------------------------------ACTACGGATACA
cDNA Xlr2*   ------------------------------------------------------------------------------------------------------------------------------------------ACTACGGATATA
```

Intron 2

```
                    380       390       400       410       420       430       440          450        460       470       480       490              500
                      *         *         *         *         *         *         *            *          *         *         *         *                *
Xlr2         CCTGTCGCTACAATCGCGAAGTAAAGAAGCGAGGTCGTCTGCCAGCCTCGGCCACAGCCAACAACAACCACAATA---CCAACGGC-AGCATCAATTCCCGAAGAGACTCTTGGCCTCTATCACACCG-ACG---A---AACAGCATACG
Xlr2-000202  CCTGTCGCTACAATCGCGAAGTAAAGAAGCGAGGTCGTCTGCCAGCCTCGGCCACAGCCAACAACAACCACAATA---CCAACGGC-AGCATCAATTCCCGAAGAGACTCTTGGCCTCTATCACACCG-ACG---A---AACAGCATACG
TAS_375582   CCTGCCGTTATAACCGCGAGGTTAAGAAACGTGGGCGTCTACCAGCCTCGGCTACAG-CA--AACAATCACAATAATAGCAATGGT-AGCATCAGCAATCGGAGAGACTCCTGGGCTTCGTCAGCTCG-ACG---AGGGAGCATCATTCG
TA_229644    CTTGTCGTTACAATCGCGAAGTAAAGAAACGAGGGCGTCTGCCGGCCTCGGCTACAG-CA--AACAATCACAAGA---GCAATGGC-AGCATCAGTAACCGGAGAGACTCGTGGGCTTCGTCGACCCG-ACG---A---GGAAGCATC--
TLONGI_14376 CCTGTCGATACAATCGCGAAGTCAAGAAGAGAGGCCGTCTACCAGCCTCGGCCACGGCCAACAACGGCAACATCAG--CGCCCGACGAGACTCTTGGCCTTTGTCAACTCGCCGCGGC-AGCATCCGGAACG---A---CTC-CCACCCG
TR_60282     CCTGTCGTTATAACCGTGAAGTCAAGAAGAGAGGCCGTCTACCGGCCTCGGCTACGGCCAACAAC------------------GGC-AACATCAGCGCCCGAAGGGAATCCTGGCCGTTGTCAACTCG-CCG---T---GGAAGCATCCG
TRICITRO_110 CCTGTCGTTACAACCGCGAGGTCAAGAAGAGAGGCCGCCTACCAGCCTCGGCCACG---GGCAACAACCAC---A---------GC-AACATCAGTGCGCGCAGAGACTCCTGGCCCTTGTCAAGTCG-CCG---A---GGCAGCATCCG
THZ_79686    CCTGTCGTTACAATCGCGAAGTCAAGAAGCGAGGTCGACTACCAGCCTCAGCCACGGCCAACAACCACAACACCA---ACGGCAGC-AGCATCAATGCCCGAAGAGACTCTTGGCCTCAATCACATCG-ACG---A---GGCAGCATTCG
TH_701679    CCTGTCGTTACAATCGCGAAGTCAAGAAGCGAGGTCGACTACCAGCCTCAGCCACGGCCAACAACCACAACACCA---ACGGCAGC-AGCATCAATGCCCGAAGAGACTCTTGGCCTCAATCACATCG-ACG---A---GGCAGCATTCG
A0A1T3C8Z2   CCTGTCGTTATAATCGCGAAGTCAAGAAGCGAGGCCGGCTGCCAGCCTCGGCCACAGCCAACAACCACAATACCA---ACGGCAGC-AGCATCAATGCCCGAAGAGACTCTTGGCCTCAATCACACCG-ACG---A---GGCAGTATTCG
TGAM_8287    CTTGTCGTTATAATCGCGAAGTAAAGAAACGAGGGCGTCTGCCGGCCTCGGCTACAG-CA-----AACCACAAGA---GCAATGGC-AGCATCAGTAATCGGAGAGACTCATGGGCTTCGTCGACCCG-ACGGGGA---AGCATCATACG
OPB37586     CCTGTCGTTATAATCGCGAAGTCAAGAAGCGAGGCCGGCTGCCAGCCTCGGCCACAGCCAACAACCACAATACCA---ACGGCAGC-AGCATCAATGCCCGAAGAGACTCTTGGCCTCAATCACACCG-ACG---A---GGCAGTATTCG
cDNA OPB3758 CCTGTCGTTATAATCGCGAAGTCAAGAAGCGAGGCCGGCTGCCAGCCTCGGCCACAGCCAACAACCACAATACCA---ACGGCAGC-AGCATCAATGCCCGAAGAGACTCTTGGCCTCAATCACACCG-ACG---A---GGCAGTATTCG
cDNA Xlr2*   CCTGTCGCTACAATCGCGAAGTAAAGAAGCGAGGTCGTCTGCCAGCCTCGGCCACAGCCAACAACAACCACAATA---CCAACGGC-AGCATCAATTCCCGAAGAGACTCTTGGCCTCTATCACACCG-ACG---A---AACAGCATACG
```

```
                         520       530                 540         550         560                           570                580             590            600
             *          *         *                   *           *           *                            *                  *               *              *
Xlr2         AAACG-ATTCAAACTTGGTGCGT--A--GC------GGCAATGGAAAC-GGTA--GCAGTAACAGCAACA------------------GCAATGGCAT---C----TC-CCAG-G-CA--TCG-TGC--TCG---TCTCCGTCAGCCA-C
Xlr2-000202  AAACG-ATTCAAACTTGGTGCGT--A--GC------GGCAATGGAAAC-GGTA--GCAGTAACAGCAACA------------------GCAATGGCAT---C----TC-CCAG-G-CA--TCG-TGC--TCG---TCTCCGTCAGCCA-C
TAS_375582   CCGTG-ATTCCAACATAGGGCAT--A--GC------GGCAATGG---------------CAATGGCAATG------------------GAAATGGTAT---C----TC-ACGG-G-CA--TCT-GTC--TCG---TCTCCATCAGCCA-C
TA_229644    ATACG-A--CGAGATTCCAACAT-----G-------GGACATGCCAGC-AATG--GCAG---CAGCAATG------------------GCAACGGCAA---C----TC-GAGA-G-CA--TCC-ATC--TCG---TCTCCATCAGCCAGT
TLONGI_14376 AGCCGCAACGCCAATGGCAACATCAACTCC------AGCAACAGCAAC-GGCC--ACGCCATGGCCAACG------------------GCATTGCCAT---C----TC-GCAG-A-CC--TCG-TCC--TCG---TCTCCGTCAGCCA-C
TR_60282     CAACG-ACTCTCCCCTGAGTCGC--A--ACGCCAATGGCAATAGCAAC-GGCA--GCAG---CAGCAACGGCCACGCCATGGGCTACGGCAATGCCAT---C----TC-GCAG-G-CC--TCG-CCC--TCG---TCTCTGTCAGCCA-C
TRICITRO_110 GAACG-ACCCCCACCTGGACCGC--A--AT------GCCAATGGCAACAGCAACTCCAACAGCAGCAACG------------------GCCACGCCATGGGCA---ACGGCAGTGTCATCTCGCAGGCTTCGCCCTCTCCGTCAGCCA-C
THZ_79686    AAACG-ATTCAATCCTAGGCCGT--A--GC------AGCAATGGCAAT-AGTA--ACAGCAACAGCAACA------------------GCAATGGCAT---C----TC-CCAG-G-CG--TCC-TGC--TCA---TCT-C--CA------
TH_701679    AAACG-ATTCAATCCTAGGCCGT--A--GC------AGCAATGGCAAT-AGTA--ACAGCAACAGCAACA------------------GCAATGGCAT---C----TC-CCAG-G-CG--TCC-TGC--TCA---TCT-C--CA------
A0A1T3C8Z2   AAACG-ATTCAAACCTGGGCCGT--A--GC------AGCAATGGCAAT-AGTA--ACAGCAACAGCAACA------------------GCAATGGCAT---C----TC-CCAG-G-CG--TCC-TGC--TC-------C--TC-TCCA--
TGAM_8287    ACGAG-AGTCTAACATAGGACAC--A--CC------AGCAATG------------GCAG---------CA------------------GCAATGGTAA---CGGCATCTCGAGAG-CA--TCT-GTC--TCG---TCTCCTTCAGCCA-G
OPB37586     AAACG-ATTCAAACCTGGGCCGT--A--GC------AGCAATGGCAAT-AGTA--ACAGCAACAGCAACA------------------GCAATGGCAT---C----TC-CCAG-G-CG--TCC-TGC--TC-------C--TC-TCCA--
cDNA OPB3758 AAACG-ATTCAAACCTGGGCCGT--A--GC------AGCAATGGCAAT-AGTA--ACAGCAACAGCAACA------------------GCAATGGCAT---C----TC-CCAG-G------CG-TCC--TG-----CTCC-TC-TCCA--
cDNA Xlr2*   AAACG-ATTCAAACTTGGTGCGT--A--GC------GGCAATGGAAAC-GGTA--GCAGTAACAGCAACA------------------GCAATGGCAT---C----TC-CCAG-G-CA--TCG-TGC--TCG---TCTCCGTCAGCCA-C

            610         620       630       640       650       660       670       680       690       700               710       720       730       740
             *           *         *         *         *         *         *         *         *         *                 *         *         *         *
Xlr2         CTCAGCT--AGCATCAGCTCTAGCACCGTTGGACAGCATCCCTTTGCCGACCCAGTGGATGCAGCCTCGGCCGGTCCCGATGGTGTAACCTTGGAT---C--GA--GATCAGGTCACCATGTCTGACACTCGCCAAGATGTGCTGCACGC
Xlr2-000202  CTCAGCT--AGCATCAGCTCTAGCACCGTTGGACAGCATCCCTTTGCCGACCCAGTGGATGCAGCCTCGGCCGGTCCCGATGGTGTAACCTTGGAT---C--GA--GATCAGGTCACCATGTCTGACACTCGCCAAGATGTGCTGCACGC
TAS_375582   CTCGGCC--AGTATCAGCTCCAGCACGGCTGCACGACGTTCGCTCACTGATCCTGTGGATGTGACCACGGCTACTACCGATGAT----------------------GATCAAATCGCTCTACAGGACTCTCGACAAGATGTGCTGCACTC
TA_229644    ATCAGCTCCAGCA------------CGGCTGCACGGCACTCACTCACTGACGCTGTGGATGTGACCACGGCTACCACCGA------------GGAT----------GATCAGATCACTCTACAAGACACTCGACAGGATGTCCTCCATTC
TLONGI_14376 CTCGCCC--AGCATCAGCTCTAGCACGGTTGCACAGCATCCCTTTGGTGACCCTACGGATGCAACCTCGGCCCGTCCCGAAGGTGTACCCCTGGATC--CCAGG--GATCGGGTTACCATGCCCGACGCTCGCCAAGACGTGCTGCATTC
TR_60282     CTCGCCC--AGCATCAGCTCTAGCACGGTCGCACAGCATCCCTTTGATGACCCAACGGATGCAACCTCGGCTCGTCCCGAAGGTGCACCCCTGGATC--C--GAGGGATCGGGTCGCCATGTCCGACGTTCGCCCAGACGTGCTGCATTC
TRICITRO_110 TTCACCC--AGCATCAGCTCCAGTACGGTTGCACAACATCCCTTTGCCGGCCCAGCGGATGCAACATCGGCCCGTCCCGAAGGTGTACCCTTGGATC--C--GAGGGATCGGGTCACCATGTCCGACGCTCGCCAAGACGTGCTGCATTC
THZ_79686    -TCGGCC--AGCATCAGCTCTAGCACCGTTGGACAGCATCCTTTTGCCGACCCGGTGGATGCAACGTCGGCCGGTCCCGAAAGTGTAACCTTGGATCAGC--GG--GATCAGGTCACCGTACCTGACGCTCGCCAAGACATGCTGCATTC
TH_701679    -TCGGCC--AGCATCAGCTCTAGCACCGTTGGACAGCATCCTTTTGCCGACCCGGTGGATGCAACGTCGGCCGGTCCCGAAAGTGTAACCTTGGATCAGC--GG--GATCAGGTCACCGTACCTGACGCTCGCCAAGACATGCTGCATTC
A0A1T3C8Z2   -TCAGCC--AGCATCAGCTCTAGCACTGTTGGACAGCATCCTTTTGCCGACACAGTGGATGCAACGTCGGCCGGTCCCGAAAGTGTAACCTTGGATCAGC--GG--GATCAGGTCACCGTACCTGACGCTCGCCAAGACATGCTGCATTC
TGAM_8287    T--ATC---AGCTCCAGCTC------GGCTGCACGCCATTCACTCACTGACGCTGTGGATGTGACCACGGCCACTACTGA------------GGAT----------GATCAGATCACTCTGCAGGACGCTCGCCAAGATGTCCTGCACTC
OPB37586     -TCAGCC--AGCATCAGCTCTAGCACTGTTGGACAGCATCCTTTTGCCGACACAGTGGATGCAACGTCGGCCGGTCCCGAAAGTGTAACCTTGGATCAGC--GG--GATCAGGTCACCGTACCTGACGCTCGCCAAGACATGCTGCATTC
cDNA OPB3758 -TCAGCC--AGCATCAGCTCTAGCACTGTTGGACAGCATCCTTTTGCCGACACAGTGGATGCAACGTCGGCCGGTCCCGAAAGTGTAACCTTGGATCAGC--GG--GATCAGGTCACCGTACCTGACGCTCGCCAAGACATGCTGCATTC
cDNA Xlr2*   CTCAGCT--AGCATCAGCTCTAGCACCGTTGGACAGCATCCCTTTGCCGACCCAGTGGATGCAGCCTCGGCCGGTCCCGATGGTGTAACCTTGGAT---C--GA--GATCAGGTCACCATGTCTGACACTCGCCAAGATGTGCTGCACGC

                         760       770       780       790       800       810       820       830       840       850       860       870       880       890
             *          *         *         *         *         *         *         *         *         *         *         *         *         *         *
Xlr2         TATCAGC-CAGCCTTACTCTCAAAGCCGGACTGCTCCTCCGCTCAGCGTCCCGTCTCTTATCCAAGTCGGACTTCAGCCGCCGCCGCCCCAGCGTTCTGCCATAGGTAATCCTTTGTCAGTCAACGTGTTTGACGGCCAGTTTGCCGACT
Xlr2-000202  TATCAGC-CAGCCTTACTCTCAAAGCCGGACTGCTCCTCCGCTCAGCGTCCCGTCTCTTATCCAAGTCGGACTTCAGCCGCCGCCGCCCCAGCGTTCTGCCATAGGTAATCCTTTGTCAGTCAACGTGTTTGACAGCCAGTTTGCCGACT
TAS_375582   CATCAGT-CAACCCTATCCTCAAAGTCGGCCAGCACCGCCGCTCAGCGTCCCGTCTCTCATCCAGGTCGGAATACAGCCACCAATGCCCCCGCGCTCCTCCATAAGCAATTCCTTGTCTGTAAATGTTTTTGAAGGACAGTTTGGTGACT
TA_229644    CATCAGC-CAGCCTTATCCTCAGAGCCGGCCAGCACCGCCGCTCAGCGTCCCGTCTCTCATCCAGGTCGGAATACAACCACAAATGCCGCCACGCTCCTCCATAAGCAACCCCCTGTCTGTCAATGTCTTTGACGGACAATTTGGAGATT
TLONGI_14376 CATCAGC-GCGCCCTTTCCTCAGAGTCGACCTGCTCCCCCGCTCAGCGTCCCGTCTCTCAGCCAAGTTGGACTCCAACCGCCGCCTGCTCAGCGTGCCTCCATAAGTCATCCCCTGTCTGTGAACGTGTTTGACGGGCAGTTTGGTGACT
TR_60282     CATCAGCGCACCCTTTC-CCCAGAGTCGACCCGCCCCTCCGCTCAGCGTCCCGTCTCTCAGCCAAGTTGGACTCCAACCGCCTTCTGCGCAGCGCGCTTCCATAAGCAACCCCCTGTCTGTGAATGTGTTTGAGGGGCAGTTTGGGGACT
TRICITRO_110 CATCAGT-GCGCCCTTTCCTCAGAGCCGACCCGCTCCTCCGCTCAGCGTCCCGTCTCTCACCCAGGTTGGACTCCAGCCGCCTCCTGCTCAGCGTGCTTCCATAAGCAACCCCCTGTCGGTGAATGTGTTTGACGGGCAGTACGGTGACT
THZ_79686    CATCAGT-CAGCCTTACTCTCAAAGTCGAACTGCTCCTCCACTCAGCGTCCCGTCTCTTATCCAAGTCGGACTTCAACCGCCACCTCCTCAGCGTTCCTCCATAGGCAATTCTCTGTCAGTCAGTGTATTTGACGGACAATTTGCTGACT
TH_701679    CATCAGT-CAGCCTTACTCTCAAAGTCGAACTGCTCCTCCACTCAGCGTCCCGTCTCTTATCCAAGTCGGACTTCAACCGCCACCTCCTCAGCGTTCCTCCATAGGCAATTCTCTGTCAGTCAGTGTATTTGACGGACAATTTGCTGACT
A0A1T3C8Z2   CATCAGT-CAGCCTTACTCTTCAGGTCGAACTGCTCCTCCACTCAGCGTCCCGTCTCTTATCCAAGTCGGACTTCAACCGCCGCCTCCCCAGCGTTCCTCCATAGGCAATTCTCTGTCAGTCAGTGTATTCGACGGACAGTTTGCGGACT
TGAM_8287    CATCAGC-CAGCCTTATCCTCAAAGTCGGCCAGCACCGCCGCTCAGCGTCCCGTCTCTCATCCAGGTCGGAATCCAGCCGCCGATGCCGCCACGCTCCTCTATAAGCAATCCCTTATCTGTGAATGTTTTCGATGGGCAATTTGGAGACA
OPB37586     CATCAGT-CAGCCTTACTCTTCAGGTCGAACTGCTCCTCCACTCAGCGTCCCGTCTCTTATCCAAGTCGGACTTCAACCGCCGCCTCCCCAGCGTTCCTCCATAGGCAATTCTCTGTCAGTCAGTGTATTCGACGGACAGTTTGCGGACT
cDNA OPB3758 CATCAGT-CAGCCTTACTCTTCAGGTCGAACTGCTCCTCCACTCAGCGTCCCGTCTCTTATCCAAGTCGGACTTCAACCGCCGCCTCCCCAGCGTTCCTCCATAGGCAATTCTCTGTCAGTCAGTGTATTCGACGGACAGTTTGCGGACT
cDNA Xlr2*   TATCAGC-CAGCCTTACTCTCAAAGCCGGACTGCTCCTCCGCTCAGCGTCCCGTCTCTTATCCAAGTCGGACTTCAGCCGCCGCCGCCCCAGCGTTCTGCCATAGGTAATCCTTTGTCAGTCAACGTGTTTGACGGCCAGTTTGCCGACT
```

```
              900       910       920       930       940       950       960       970       980       990      1000      1010      1020      1030      1040
                *         *         *         *         *         *         *         *         *         *         *         *         *         *         *
Xlr2          CCGAAGTCGTCTTGTCTGATGAAAGCGCTGGCTATCCGTCACCGGTCAACGGCCGGCCCCATATCGAGGCTGGAAATACGCGCCAGCACCGAGGCTCTTTCAAGTCGACTCCCAGTCATGAACGAAACCGAGAGTCTTTTAGCACCGCCG
Xlr2-000202   CCGAAGTCGTCTTGTCTGATGAAAGCGCTGGCTATCCGTCACCGGTCAACGGCCGGCCCCATATCGAGGCTGGAAATACGCGCCAGCACCGAGGCTCTTTCAAGTCGACTCCCAGTCATGAACGAAACCGAGAGTCTTTTAGCACCGCCG
TAS_375582    CGGAAGTTGCCTTGTCTGACGGAAGTGTTGGCTATCCATCACCAGTCGACAGGCGACCCCATGTTGAGGCCGCCAATTCACGACAACACAGAGGCTCTTTCAAGTCAACTCCCAGCATTGAGCGGAACCGAGAGTCATTCAGCAC-----
TA_229644     CTGAAGTTGCCCTGTCTGACGGAAGCGTTGGTTACCCATCGCCAATCGACAGACGGCCCCATGCCGAGGCCCCCAACGCACGCCAACACCGAGGCTCGTTCAAGTCCACCCCCAGCCATGAACAGAACCGCGGGTCGTTCAGCAC-----
TLONGI_14376  CCGAAGTCGTCTTGTCCGATGAGAGCGTTGGCTACCCGTCACCGGTCAACACCCGGCCTCATGCTGAGGT---AA--AC-CGCCAACACAGGGCGTCGTTCAAGTCGACGCACAGCCATGAGCGGAACAGAGAGTCGTTCAGCACGGCGG
TR_60282      CGGAAGTCGTCGTGTCCGATGAGAGCGTTGGATACCCGTCACCGATAAACACCAGGCCCCATGCCGA-GCT-GA---AC-CGCCAACACAGGGCGTCCGGCAAGTCGACGCACAGCCACGAGCGGAACAGAGAGTCATTCAGCACAGCGG
TRICITRO_110  CCGAAGTCATCCTGTCCGATGAGAGTGCTGGCTACCCGTCACCGGTCAACACCCGGCCCCATGCCGAGG-TG--A--AC-CGCCAGCACAGGGCATCTTTCAAGTCGACGCACAGCCATGAGCGGAACAGAGAGTCGTTCAGCACAGCAG
THZ_79686     CAGAGGTCGTCCTGTCTGATGAAAGCGCTGGCTACCCCTCGCCAGTAAACGGACGGCCTCCTATCGAGACTGGAAGTGCGCGCCAGCACAGAGGCTCCTTTAAATCGACTCCCAGTCATGAACGAAACCGAGAGTCGTTCAGCACTGCTG
TH_701679     CAGAGGTCGTCCTGTCTGATGAAAGCGCTGGCTACCCCTCGCCAGTAAACGGACGGCCTCCTATCGAGACTGGAAGTGCGCGCCAGCACAGAGGCTCCTTTAAATCGACTCCCAGTCATGAACGAAACCGAGAGTCGTTCAGCACTGCTG
A0A1T3C8Z2    CAGAGGTCGTCCTGTCTGATGAAAGCGCTGGCTATCCCTCACCAGTTAACGGACGGGCACCTATCGAGGCTGGAAGTGCACGCCAGCACAGAGGCTCCTTCAAGTCGACTCCCAGTCATGAACGGAACCGAGAGTCGTTTAGCACTGCTG
TGAM_8287     ATGAAGTTGCCTTGTCTGACGGAAGCGTTGGCTATCCATCGCCAGTCGACAGACGGTCCCATGCCGAGGCCGCCAATGCCAGACAACACCGAGGCTCTTTCAAGTCTACTCCCAGCCATGAACGGAACCGAGAGTCATTCAGCA---C--
OPB37586      CAGAGGTCGTCCTGTCTGATGAAAGCGCTGGCTATCCCTCACCAGTTAACGGACGGGCACCTATCGAGGCTGGAAGTGCACGCCAGCACAGAGGCTCCTTCAAGTCGACTCCCAGTCATGAACGGAACCGAGAGTCGTTTAGCACTGCTG
cDNA OPB3758  CAGAGGTCGTCCTGTCTGATGAAAGCGCTGGCTATCCCTCACCAGTTAACGGACGGGCACCTATCGAGGCTGGAAGTGCACGCCAGCACAGAGGCTCCTTCAAGTCGACTCCCAGTCATGAACGGAACCGAGAGTCGTTTAGCACTGCTG
cDNA Xlr2*    CCGAAGTCGTCTTGTCTGATGAAAGCGCTGGCTATCCGTCACCGGTCAACGGCCGGCCCCATATCGAGGCTGGAAATACGCGCCAGCACCGAGGCTCTTTCAAGTCGACTCCCAGTCATGAACGAAACCGAGAGTCTTTTAGCACCGCCG
```

```
         1050         1060      1070      1080      1090      1100      1110      1120      1130      1140      1150      1160      1170      1180      1190
            *           *         *         *         *         *         *         *         *         *         *         *         *         *         *
Xlr2          CAAC-TG-C---CCATGATTCAGGGGCCCTCATGTTGGACTTCTTCCACAAGGCCCCAAACGAGGATTGCTGGTACAAGTTTCTCGAGCCGATCTTGCCGTACATCCGCAACATCATTCCCGCCTCTGTTGCGTGTGATTTACTCGACAT
Xlr2-000202   CAAC-TG-C---CCATGATTCAGGGGCCCTCATGTTGGACTTCTTCCACAAGGCCCCAAACGAGGATTGCTGGTACAAGTTTCTCGAGCCGATCTTGCCGTACATCCGCAACATCATTCCCGCCTCTGTTGCGTGTGATTTACTCGACAT
TAS_375582    AAAC-AG-----CCACGATGCAGGAGGCCTGATGCTGGATTTCTTCCACAAGGCGCCCAACGACGACTGTTGGTACAAGTTTCTTGAGCCAATCTTGCCCTACATCCGTAACATCATTCCCGCCTCTGTTGCGTGCGACTTGCTGGAGAT
TA_229644     AAAC-AG-----CCACGATGCAGGAGGCTTGATGCTGGATTTCTTCCACAAGGCGCCAAACGACGACTGCTGGTACAAGTTCCTCGAGCCAATTCTGCCCTACATCCGCAACATCATCCCCGCCTCCGTTGCGTGCGACTTGCTGGAAAT
TLONGI_14376  CAACAAC-CAA-CCACGATGCCGGGGGCCTGATGCTGGATTTCTTCCACAAGGCTCCCAACGAGGACTGCTGGTACAAGTTCCTGGAGCCCATCTTGCCGTACATCCGCAACATCATCCCCGCCTCTGTTGCCTGCGATTTGCTCGACAT
TR_60282      CAAC-GA-CCAGCCACGATGTCGGGGGCCTGATGTTGGATTTCTTCCACAGAGCCCCAAACGAGGACTGCTGGTACAAGTTTCTGGAGCCCATCTTGCCGTACATCCGCAACATCATCCCCGCCTCTGTTGCGTGCGACTTGCTCGACAT
TRICITRO_110  CAAC-AGCCAG-CCATGATGCCGGGGGCCTGATGTCGGATTTCTTCCACAAGGCTCCCAACGAGGACTGCTGGTACAAGTTTCTGGAGCCCATCTTGCCATACATCCGCAACATCATCCCCGCCTCTGTCGCCTGCGATTTGCTCGACAT
THZ_79686     CAAC-TG-G---CCATGATGCCGCGGGTCTCATGTTGGACTTCTTTCACAAGGCCCCAAACGAGGATTGCTGGTACAAGTTTCTTGAGCCAATCTTGCCATACATCCGCAACATCATTCCCGCCTCTGTTGCATGCGATTTGCTCGACAT
TH_701679     CAAC-TG-G---CCATGATGCCGCGGGTCTCATGTTGGACTTCTTTCACAAGGCCCCAAACGAGGATTGCTGGTACAAGTTTCTTGAGCCAATCTTGCCATACATCCGCAACATCATTCCCGCCTCTGTTGCATGCGATTTGCTCGACAT
A0A1T3C8Z2    CGAC-TG-G---CCACGATGCCGCGGGACTCATGTTGGACTTCTTCCACAAGGCCCCAAACGAGGATTGTTGGTACAAGTTTCTTGAGCCAATCTTGCCATACATCCGCAACATCATTCCGGCCTCTGTTGCATGCGATCTGCTCGACAT
TGAM_8287     AAAC-AG-----CCACGACGCAGGAGGCTTGATGCTGGACTTCTTCCACAAAGCGCCAAACGACGACTGCTGGTACAAGTTTCTCGAGCCAATCCTGCCGTATATCCGCAACATCATTCCTGCCTCTGTTGCGTGCGACTTGCTGGAAAT
OPB37586      CGAC-TG-G---CCACGATGCCGCGGGACTCATGTTGGACTTCTTCCACAAGGCCCCAAACGAGGATTGTTGGTACAAGTTTCTTGAGCCAATCTTGCCATACATCCGCAACATCATTCCGGCCTCTGTTGCATGCGATCTGCTCGACAT
cDNA OPB3758  CGAC-TG-G---CCACGATGCCGCGGGACTCATGTTGGACTTCTTCCACAAGGCCCCAAACGAGGATTGTTGGTACAAGTTTCTTGAGCCAATCTTGCCATACATCCGCAACATCATTCCGGCCTCTGTTGCATGCGATCTGCTCGACAT
cDNA Xlr2*    CAAC-TG-C---CCATGATTCAGGGGCCCTCATGTTGGACTTCTTCCACAAGGCCCCAAACGAGGATTGCTGGTACAAGTTTCTCGAGCCGATCTTGCCGTACATCCGCAACATCATTCCCGCCTCTGTTGCGTGTGATTTACTCGACAT
```

```
                  1200      1210      1220      1230      1240      1250      1260      1270      1280      1290      1300      1310      1320      1330      1340
                     *         *         *         *         *         *         *         *         *         *         *         *         *         *         *
Xlr2          CTTCTTGACCGACCCGGGGAGCTCGCTGTTTCGCGTCGCATCTCCATATATATTGACCAGAGTCTTTCGTAAAAAGTCCATTATACATCCCACAGCTCCCAGATATACCACACCGGCACTGCTTGCAACAATCCTGTGGTGTGTTGCACA
Xlr2-000202   CTTCTTGACCGACCCGGGGAGCTCGCTGTTTCGCGTCGCATCTCCATATATATTGACCAGAGTCTTTCGTAAAAAGTCCATTATACATCCCACAGCTCCCAGATATACCACACCGGCACTGCTTGCAACAATCCTGTGGTGTGTTGCACA
TAS_375582    CTTCCTGACGGACCCGGGAAGTTCACTGTTTCGCGTTGCTTCTCCGTATATATTGACCAGAGTCTTCCGCAAGAAGTCAATTACGCATCCGACCAACCCCCGATATACCACGCCCGCGTTGCTTGCGACAATTTTGTGGTGTGTGGCGCA
TA_229644     CTTCCTGACGGATCCCGGAAGCTCGCTTTTTCGCGTTGCTTCGCCGTACATCTTGACCAGAGTCTTTCGCAAAAAGTCGATTATGCATCCGACGAACCCTCGATATACCACGCCTGCATTGCTCGCGACGATTCTGTGGTGCGTGGCGCA
TLONGI_14376  ATTCCTGACGGACCCGGGGAGCTCGCTGTTTCGCGTTGCGTCCCCGTATATACTGACAAGAGTCTTTCGTAAGAAATCTATTGTGCATCCGACGAATCCGAGATACACCACCCCGGCCCTGCTTGCGACCATACTGTGGTGTGTAGCACA
TR_60282      ATTTCTGACGGATCCGGGAAGCTCGCTGTTTCGTGTTGCGTCTCCCCTATATCCTGACTAGGGTCTTTCGTAAAAAGTCCATTGTGCACCCGACGAACCCCAGATACACCACCCCGGCGCTGCTGGCGACCATACTCTGGTGTGTAGCACA
TRICITRO_110  TTTCCTGACGGACCCGGGGAGCTCGCTGTTTCGCGTTGCGTCTCCGTATATCTTGACCAGAGTCTTTCGTAAAAAGTCTATCGTGCACCCGACGAACCCCAGATACACCACCCCGGCTCTGCTGGCGACCATACTCTGGTGTGTAGCACA
THZ_79686     CTTCTTGACCGACCCGGGGAGCTCCTTGTTTCGTGTCGCATCTCCATACATCTTGACCAGAGTCTTTCGTAAAAAGTCTATTGTACATCCAACAAACCCCAGATATACCACACCAGCGTTGTTGGCAACAATCCTTTGGTGTGTAGCACA
TH_701679     CTTCTTGACCGACCCGGGGAGCTCCTTGTTTCGTGTCGCATCTCCATACATCTTGACCAGAGTCTTTCGTAAAAAGTCTATTGTACATCCAACAAACCCCAGATATACCACACCAGCGTTGTTGGCAACAATCCTTTGGTGTGTAGCACA
A0A1T3C8Z2    TTTCCTGACCGATCCGGGGAGCTCCTTGTTTCGTGTCGCATCTCCCTACATTTTAACCAGAGTCTTTCGTAAAAAGTCTATCGTACATCCAACAAATCCCAGATATACCACACCGGCGTTGTTGGCAACAATCCTATGGTGTGTAGCACA
TGAM_8287     CTTCCTGACGGATCCCGGAAGTTCGCTTTTTCGCGTTGCTTCGCCATATATTTTGACCAGAGTCTTTCGCAAAAAGTCGATTACGCATCCGACCAACCCTCGATATACCACGCCTGCATTGCTTGCGACGATTCTGTGGTGCGTGGCGCA
OPB37586      TTTCCTGACCGATCCGGGGAGCTCCTTGTTTCGTGTCGCATCTCCCTACATTTTAACCAGAGTCTTTCGTAAAAAGTCTATCGTACATCCAACAAATCCCAGATATACCACACCGGCGTTGTTGGCAACAATCCTATGGTGTGTAGCACA
cDNA OPB3758  TTTCCTGACCGATCCGGGGAGCTCCTTGTTTCGTGTCGCATCTCCCTACATTTTAACCAGAGTCTTTCGTAAAAAGTCTATCGTACATCCAACAAATCCCAGATATACCACACCGGCGTTGTTGGCAACAATCCTATGGTGTGTAGCACA
cDNA Xlr2*    CTTCTTGACCGACCCGGGGAGCTCGCTGTTTCGCGTCGCATCTCCATATATATTGACCAGAGTCTTTCGTAAAAAGTCCATTATACATCCCACAGCTCCCAGATATACCACACCGGCACTGCTTGCAACAATCCTGTGGTGTGTTGCACA
```

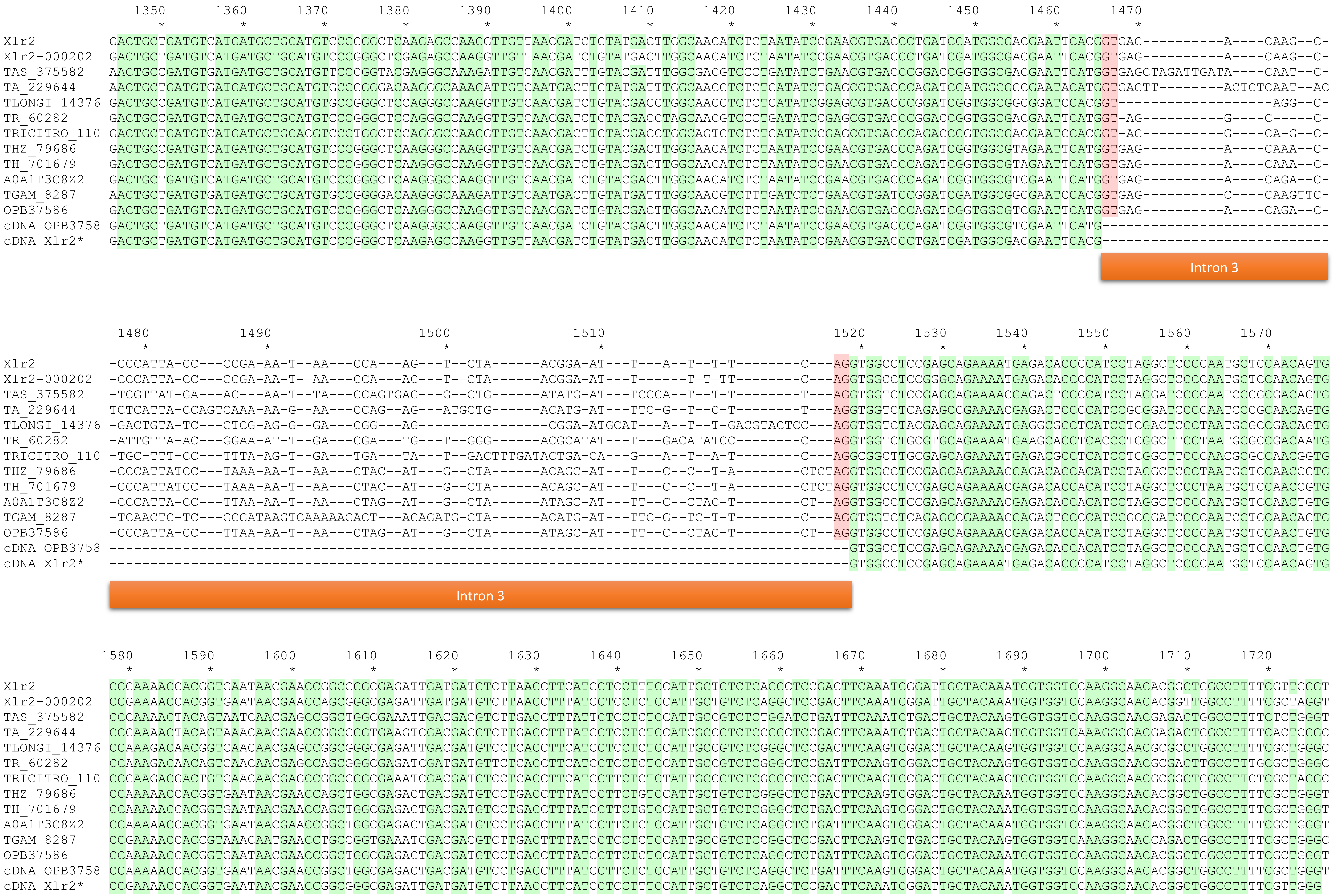

1350 1360 1370 1380 1390 1400 1410 1420 1430 1440 1450 1460 1470
Xlr2
Xlr2-000202
TAS_375582
TA_229644
TLONGI_14376
TR_60282
TRICITRO_110
THZ_79686
TH_701679
A0A1T3C8Z2
TGAM_8287
OPB37586
cDNA OPB3758
cDNA Xlr2*
Intron 3
1480 1490 1500 1510 1520 1530 1540 1550 1560 1570
Intron 3
1580 1590 1600 1610 1620 1630 1640 1650 1660 1670 1680 1690 1700 1710 1720

```
                1730      1740      1750      1760      1770      1780      1790      1800      1810      1820      1830      1840      1850      1860      1870
                   *         *         *         *         *         *         *         *         *         *         *         *         *         *         *
Xlr2            CTCAATCGAGAGGATGAACAATGTGCCGGACCTGTGACACCATGTGCAAACCCCCTTTGCTCGTGCAAAAAGGACCAAGATGGCAGCATATACAATATGGAGTTACGGGAAGAACGCCGGAGAGTCTTTTGGCTGCTCTACGCCTTGGAT
Xlr2-000202     CTCAATCGAGAGGATGAGCAATGTGCCGGACCTGTGACGCCATGTGCAAACCCC-TTTGCTCGTGCAAAAAGGACCAAGATGGCAGCATATACAATATGGAGTTGCGGGAAGAACGCCGGAGAGTCTTTTGGCTGCTCTACGCCTTGGAT
TAS_375582      CTCAATCGAGAAGACGAGCAGTGTGCTGGACCTGTAACTCCATGCGCAAATCCCCTCTGCTCATGTCGAAAGGAGCAGGACGGCAGCTTATACAATCTCGAGCATCGAGAGGAACGCCGGAGAGTCTTTTGGCTTCTCTACGCTCTGGAC
TA_229644       CTCAATCGAGAAGACGAGCAGTGTGCCGGGCCCGTAACTCCTTGCGCGAATCCCCTCTGCTCATGTCGAAAGGAGCAGGACGGCAGCTTGTACAATGTGGAGCATCGAGAGGAACGCAGGAGAGTCTTTTGGCTTCTGTACGCTCTGGAT
TLONGI_14376    CTCAACCGGGAGGATGAGCAGTGCGCCGGGCCCGTAACGCCTTGCGCAAACCCTCTCTGCTCATGTCGTAAGGAGCAGGATGGCAGCCTGTATAACCTCGAGCTGCGAGAAGAGCGCCGGCGGGTCTTTTGGCTTCTCTATGCCCTGGAT
TR_60282        CTCAACCGAGAGGACGAGCAATGCGCCGGACCCGTGACGCCCTGCGCGAATCCTCTCTGCTCGTGTCGAAAAGAGCAGGATGGTAGCTTGTACAATCTCGAGCTGCGAGAAGAGCGCCGGCGAGTCTTTTGGCTTCTCTATGCCTTGGAC
TRICITRO_110    CTCAACCGGGAGGATGAACAGTGCGCCGGGCCCGTAACGCCGTGCGCAAACCCTCTCTGCTCATGTCGAAAAGAGCAAGATGGTAGTCTGTATAATCTCGAGCTGCGAGAAGAGCGCCGAAGAGTCTTTTGGCTTCTCTATGCCCTGGAC
THZ_79686       CTACACCGAGAGGACGAGCAATGTGCCGGGCCTGTGACGCCGTGCGCAAACCCCCTTTGCTCGTGCAAAAAAGACCAAGATGGCAGCATATATAACTTGGAGCTACGAGAAGAGCGTCGAAGAGTCTTTTGGCTTCTCTACGCTCTGGAT
TH_701679       CTACACCGAGAGGACGAGCAATGTGCCGGGCCTGTGACGCCGTGCGCAAACCCCCTTTGCTCGTGCAAAAAAGACCAAGATGGCAGCATATATAACTTGGAGCTACGAGAAGAGCGTCGAAGAGTCTTTTGGCTTCTCTACGCTCTGGAT
A0A1T3C8Z2      CTACACCGAGAGGATGAGCAATGTGCCGGACCTGTGACCCCTTGCGCAAACCCCCTTTGCTCATGCAAAAAGGATCAAGATGGCAGCATATATAACTTGGAGCTACGAGAAGAGCGCCGAAGAGTCTTTTGGCTTTATACGCCCTGGAT
TGAM_8287       CTCAATCGAGAAGACGAGCAATGCGCTGGACCCGTAACTCCTTGTGCAAATCCTCTTTGTTCATGCCGAAAGGAGCAGGATGGCAGCTTGTACAATCTGGAGCATCGTGAGGAACGCAGGAGAGTCTTTTGGCTTCTGTACGCTCTGGAT
OPB37586        CTACACCGAGAGGATGAGCAATGTGCCGGACCTGTGACCCCTTGCGCAAACCCCCTTTGCTCATGCAAAAAGGATCAAGATGGCAGCATATATAACTTGGAGCTACGAGAAGAGCGCCGAAGAGTCTTTTGGCTTTATACGCCCTGGAT
cDNA OPB3758    CTACACCGAGAGGATGAGCAATGTGCCGGACCTGTGACCCCTTGCGCAAACCCCCTTTGCTCATGCAAAAAGGATCAAGATGGCAGCATATATAACTTGGAGCTACGAGAAGAGCGCCGAAGAGTCTTTTGGCTTTATACGCCCTGGAT
cDNA Xlr2*      CTCAATCGAGAGGATGAACAATGTGCCGGACCTGTGACACCATGTGCAAACCCCCTTTGCTCGTGCAAAAAGGACCAAGATGGCAGCATATACAATATGGAGTTACGGGAAGAACGCCGGAGAGTCTTTTGGCTGCTCTACGCCTTGGAT

                1880      1890      1900      1910      1920      1930      1940            1950                      1960            1970                1980
                   *         *         *         *         *         *         *               *                       *               *                   *
Xlr2            CGCCATCTAGCCTTGTCCTTCAACTCGGCTCTAACGATACCCGATTCATACTGCGAAGTTTATTGTTAGT--A--T--A--C--CA-A--T----T--CGTT-C--T-TGA-CTG-GCTT-T-----TGTT---CTAACTT-T--T--G-
Xlr2-000202     CGCCATCTAGCCTTGTCCTTCAACTCGGCTCTAACGATACCCGATTCATACTGCGAAGTTTATTGTTAGT--A--T--A--C--CA-A--T----T--CGTT-C--T-TGA-CTG-GCTT-T-----TGTT---CTAACTT-T--T--G-
TAS_375582      AGGCATCTGGCTTTGTCCTTTAACTCGGCTTTGACAATCCCCGATTCTTACTGTGAAGTTTATTGTAAGTGGA--CTGA--G--TA-A--C----C--CATT-C--TGCTT-TTGTGCTTGA-----TTAA---CCAACCT-T--T--G-
TA_229644       CGGCATCTGGCCTTGTCTTTCAACTCGGCGTTGACGATACCTGATTCTTACTGCGAAGTCTATTGTAAGTA-ACTT--AGGC--TA-A--GTAAAT--TGTTAA--TATGT-TCT-GCTT-T-----CGTTAAACTAAC-C-C--T--G-
TLONGI_14376    CGGCATTTGGCCTTGTCTTTCAACTCTGCTCTCACAATCCCTGACTCGTACTGTGAGGTTTACTGTAAGT--A--T-----C--CA-A-------G--CCTG-C--T-TCA-GCA-GCTTCT-----TCTT---CACTCTT-T--CTAA-
TR_60282        CGGCACTTGGCCCTGTCTTTCAACTCTGCCCTTACCATCCCTGACTCGTACTGCGAAGTCTACTGTAAGG--A--C--C--A--AA-A--C----C--TGCC-C--C-CAATCTG-TTCA-T-----TGTC---CACCCTT-TGCT--GA
TRICITRO_110    CGGCACCTGGCCTTGTCTTTCAACTCTGCTCTCACAATTCCTGACTCGTACTGCGAAGTTTACTGTAAGT-----T--T--C--CA-AACA----T--TGCT-C--T-AAC-CTGTTCAT-T-----TGATTA-CTCCCTTCT--A--A-
THZ_79686       CGCCATCTAGCCTTGTCTTTCAACTCGGCTCTAACAATACCCGACTCTTATTGCGAAGTTTATTGTAAGT--A--T--A--C--TATA--TGA--TG-CCTC-CAGC-TTA-TTG-ACTG-G-----CCTTG--CTAA-TT-C--T--G-TH_701679
CGCCATCTAGCCTTGTCTTTCAACTCGGCTCTAACAATACCCGACTCTTATTGCGAAGTTTATTGTAAGT--A--T--A--C--TATA--TGA--TG-CCTC-CAGC-TTA-TTG-ACTG-G-----CCTTG--CTAA-TT-C--T--G-
A0A1T3C8Z2      CGCCATCTAGCTTTGTCTTTCAACTCGGCTCTAACAATACCTGACTCTTATTGTGAAGTTTATTGTAAGC--A--T--A--C--TA-T--G----T--TGTG-CC-T-TCA-CCC-TCCG-ACCAACTGTTG--CTAA-TT-C--T--A-
TGAM_8287       CGGCATCTGGCCTTGTCTTTCAACTCGGCGCTGACAATACCTGATTCATACTGCGAAGTCTATTGTAAGT--ACCT--AG-ACTGA-A--TG---ATCCGTT-C--T-G---CTT-TC-C-A-----TAAA---CTAAC-C-T--T--G-
OPB37586        CGCCATCTAGCTTTGTCTTTCAACTCGGCTCTAACAATACCTGACTCTTATTGTGAAGTTTATTGTAAGC--A--T--A--C--TA-T--G----T--TGTG-CC-T-TCA-CCC-TCCG-ACCAACTGTTG--CTAA-TT-C--T--A-
cDNA OPB3758    CGCCATCTAGCTTTGTCTTTCAACTCGGCTCTAACAATACCTGACTCTTATTGTGAAGTTTATT----------------------------------------------------------------------------------------
cDNA Xlr2*      CGCCATCTAGCCTTGTCCTTCAACTCGGCTCTAACGATACCCGATTCATACTGCGAAGTTTATT----------------------------------------------------------------------------------------
                                                                Intron 4

                     1990        2000      2010      2020      2030      2040      2050      2060      2070      2080      2090      2100      2110      2120      2130
                        *           *         *         *         *         *         *         *         *         *         *         *         *         *         *
Xlr2            -C--T--C--TCAGCTCCGCTACCTGAAGCTGTTTGGGAAAACCTTGATACAATCCCGCCCAGCGAAATCCCCGCTCGAAGGATCGGACCGCCAACTACCGCCTCTGGATCTGCCTTTTTTGAATACTTTTTGCCCCTCATGGCGATACT
Xlr2-000202     -C--T--C--TCAGCTCCGCTACCTGAAGCTGTTTGGGAAAACCTCGATACAATCCCGCCCAGCGAAATCCCCGCTCGAAGGATCGGACCGCCAACAACCGCCTCTGGATCTGCCTTTTTTGAATACTTTTTGCCCCTCATGGCGATACT
TAS_375582      -C--A--A--CCAGCCCCTCTGCCTGAAGCTGTTTGGGAAAACCTCGACACAATTCCTCCAAACGAGATTCCCGTTCGAACAATTGGACCACCAACGACTGCCTCCGGATCTGCCTTTTTCGAATACTTCTTACCACTCATGGCTATACT
TA_229644       -T--T--CA-TCAGCCCCCCTACCTGAAGCTATTTGGGAGAATCTCGACACCATTCCTCCCAACGAGATGCCCGTTCGCACCATTGGACCGCCAACAACGGCCTCTGGATCTGCCTTTTTCGAATACTTTTTGCCACTCATGGCTATACT
TLONGI_14376    -CA-A--GCTTCAGCCCCGCTTCCTGAAGCCATATGGGAGAACCTCGACACGATCCCACCCAGCGAAATCCCCGCTCGAAGAATAGGACCGCCAACGACTGCCTCTGGATCTGCCTTTTTTGAGTACTTCTTACCTCTCATGGCGATACT
TR_60282        CCGGC--C--TCAGCACCTCTTCCCGAAGCCATCTGGGAGAACCTCGACACGATTTCGCCCAGTGAGATTCCCGCCCGAAGAATCGGACCGCCCACGACTGCCTCTGGATCTGCATTTTTCGAGTACTTTTTACCCCTCATGGCGATACT
TRICITRO_110    -C--TGACT-TCAGCCCCCCTCCCCGAAGCCATCTGGGAAAACCTCGACACGATCCCACCCAGCGAGATTCCTGCCCGAACAATCGGACCGCCGACGACTGCCTCTGGGTCTGCATTTTTTGAGTACTTTCTACCCCTCATGGCGATACT
THZ_79686       -C--T--C--TTAGCCCCGTTGCCTGAAGCTGTTTGGGAAAACCTCGATACAATCCCACCTAGCGAAATCCCCGCCCGAAGGATCGGACCGCCAACGACTGCCTCTGGGTCTGCCTTTTTCGAATACTTTTTGCCACTCATGGCCATACT
TH_701679       -C--T--C--TTAGCCCCGTTGCCTGAAGCTGTTTGGGAAAACCTCGATACAATCCCACCTAGCGAAATCCCCGCCCGAAGGATCGGACCGCCAACGACTGCCTCTGGGTCTGCCTTTTTCGAATACTTTTTGCCACTCATGGCCATACT
A0A1T3C8Z2      -C--T--C--TCAGCCCCGTTGCCTGAAGCTGTTTGGGAAAACCTCGATACAATCCCACCCAGCGAAATCCCCGCTCGAAGGATCGGACCGCCAACGACTGCCTCTGGGTCTGCCTTTTTCGAATACTTTTTGCCGCTCATGGCGATACT
TGAM_8287       -T--T--CA-TCAGCCCCCCTACCTGAAGCTGTTTGGGAAAATCTCGACACCATTCCTCCCAACGAGATGCCCGTTCGAACCATTGGACCACCAACAACAGCCTCTGGATCTGCCTTTTTCGAATATTTCTTACCGCTCATGGCTATACT
OPB37586        -C--T--C--TCAGCCCCGTTGCCTGAAGCTGTTTGGGAAAACCTCGATACAATCCCACCCAGCGAAATCCCCGCTCGAAGGATCGGACCGCCAACGACTGCCTCTGGGTCTGCCTTTTTCGAATACTTTTTGCCGCTCATGGCGATACT
cDNA OPB3758    --------------CCCCGTTGCCTGAAGCTGTTTGGGAAAACCTCGATACAATCCCACCCAGCGAAATCCCCGCTCGAAGGATCGGACCGCCAACGACTGCCTCTGGGTCTGCCTTTTTCGAATACTTTTTGCCGCTCATGGCGATACT
cDNA Xlr2*      --------------CTCCGCTACCTGAAGCTGTTTGGGAAAACCTTGATACAATCCCGCCCAGCGAAATCCCCGCTCGAAGGATCGGACCGCCAACTACCGCCTCTGGATCTGCCTTTTTTGAATACTTTTTGCCCCTCATGGCGATACT
                  Intron 4
```

```
                2140      2150      2160      2170      2180      2190      2200      2210      2220      2230      2240      2250      2260      2270      2280
                   *         *         *         *         *         *         *         *         *         *         *         *         *         *         *
Xlr2           GGGCGACATCATAGAAGTCCACCATAGGCGCCGTCATCCAAGGCTTGGAGATCTCGACGATTCACATTCTGTAGCTGTTATTCAGGGGCTTCTTTCGACCTACGAGATGAGTCTTGCCGCGCTTTCGTCGGAGGGTAATGACAGCAACAT
Xlr2-000202    GGGCGACATCATAGAAGTCCACCATAGGCGCCGTCATCCAAGGCTTGGAGATCTCGACGATTCACATTCTGTAGCTGTTATTCAGGGGCTTCTTTCGACCTACGAGATGAGTCTTGCCGCGCTTTCGTCGGAGGGTAATGACAGCAACAT
TAS_375582     AGGAGACATTATAGAAGTCCATCACAGACGCCGCCATCCAAGACTTGGAGATCTGGACGATTCGCACTCCGTATCTATTATCCAAGGACTACTTACGACGTACGAACTGAGTCTTGCGGCGCTTTCATCAGAAGGAAATGACAACGGCAT
TA_229644      TGGAGACATCATAGAGGTCCATCACAGGCGCCGTCATCCACGGCTCGGAGATTTAGACGATTCACACTCCGTGTCTGTCATCCAGGGGCTTCTGACAACATACGAACTGAGTCTCGCGGCACTCTCGTCAGAAGGCAACGACAGTGGCAT
TLONGI_14376   AGGCGACATCATCGAAGTCCATCATCGGCGCCGTCACCCGAGACTCGGAGGTCTCGACGACTCACACTCCGTGGCCGTCATTCAGGGGCTGCTTTCGACCTACGAAATGAGTCTCGCCGCGCTTTCGCCCGACGGCGATGACAACGGCAC
TR_60282       AGGCGACATTATAGAGGTCCATCATCGGCGCCGTCACCCGAGACTCGGAGGCCTCGACGATTCACACTCCGTGGCCGTCGTTCAGGGGCTGCTTTCGGCCTACGAAATGAGTCTCGCCGCGCTGTCACCAGACGGCGACGACAACGGCAC
TRICITRO_110   GGGCGACATCATAGAAGTTCATCATCGGCGCCGTCATCCGAGACTCGGAGGCCTCGACGATTCACACTCTGTAGCCGTAATCCAGGGACTGCTTTCGACCTACGAGTTGAGTCTCGCCGCGCTTTCACCAGACGGCGACGACAACGGCAC
THZ_79686      GGGCGACATTATCGAAGTCCACCACAGACGCCGTCATCCAAGACTGGCAGGTCTCGACGACTCGCACTCTGTAGCCGTTATCCAGGAGCTCCTTTCCACCTACGAGCTCAGTCTTGCCGCTCTTTCACCAGAGAGCAATGATAACAACAC
TH_701679      GGGCGACATTATCGAAGTCCACCACAGACGCCGTCATCCAAGACTGGCAGGTCTCGACGACTCGCACTCTGTAGCCGTTATCCAGGAGCTCCTTTCCACCTACGAGCTCAGTCTTGCCGCTCTTTCACCAGAGAGCAATGATAACAACAC
A0A1T3C8Z2     GGGTGACATTATAGAAGTCCACCACAGGCGCCGTCATCCAAGACTGGCAGGTCTCGACGACTCGCACTCTGTAGCCGTTATCCAGGAACTCCTTTCCACCTACGAGCTCAGTCTTGCCGCTCTTTCGCCAGAGAGCAATGATAGCAGCAC
TGAM_8287      AGGAGACATCATAGAGGTTCATCACAGGCGCCGTCACCCACGCCTTGGAGATTTAGACGATTCACACTCCGTGTCTGTCATCCAAGGGCTTCTCACAACATACGAATTGAGTCTCGCGGCACTCTCGTCAGAAGGCAACGACAGCGGCAT
OPB37586       GGGTGACATTATAGAAGTCCACCACAGGCGCCGTCATCCAAGACTGGCAGGTCTCGACGACTCGCACTCTGTAGCCGTTATCCAGGAACTCCTTTCCACCTACGAGCTCAGTCTTGCCGCTCTTTCGCCAGAGAGCAATGATAGCAGCAC
cDNA OPB3758   GGGTGACATTATAGAAGTCCACCACAGGCGCCGTCATCCAAGACTGGCAGGTCTCGACGACTCGCACTCTGTAGCCGTTATCCAGGAACTCCTTTCCACCTACGAGCTCAGTCTTGCCGCTCTTTCGCCAGAGAGCAATGATAGCAGCAC
cDNA Xlr2*     GGGCGACATCATAGAAGTCCACCATAGGCGCCGTCATCCAAGGCTTGGAGATCTCGACGATTCACATTCTGTAGCTGTTATTCAGGGGCTTCTTTCGACCTACGAGATGAGTCTTGCCGCGCTTTCGTCGGAGGGTAATGACAGCAACAT

                2290      2300      2310      2320      2330          2340          2350      2360      2370      2380      2390      2400      2410      2420
                   *         *         *         *         *         *         *         *         *         *         *         *         *         *         *
Xlr2           AATACCTCTCCCTATTCACACCCCAAAAGGCATTGTTGCTGGAATGCCTTCAAGGC---CCT--C--CAT-C-TCTCACCCGCACCCATCATCTGCAGCGCCATACAATGCGGGTGATCCTTCCAAGATGCGGCTCGCAAAAGCCTATAG
Xlr2-000202    AATTCCCCTCCCTATTCACACCCCAAAAGGCATTGTTGCTGGAATGCCTTCAAGGC---CCT--C--CAT-C-TCTCACCCGCACCCATCATCTGCAGCGCCATACAATGCGGGCGATCCTTCCAAGATGCGGCTCGCAAAAGCCTATAG
TAS_375582     AGTTTCCCTGCCTATCCACACTCCAAAGGGCATGGTTGCCGGCATGCCGTCGAGGTCATCCATGTCTCATCCATCTCATCCATACCCACCTTCTACAGCGCCATACAACGCAGGTGATCCCTCCAAGATGCGCCTAGCCAAGGCCTATAG
TA_229644      AGTTCCCCTGCCCATCCACACGCCAAGGGGCATGGTCGCCGGCATGCCTTCGCGGT---CAT--C--CAT-G-TCCCATCCGTATCCGCCCTCCACAGCGCCATACAACGCAAGCGATCCGTCCAAGATGCGCCTCGCCAAGGCCTACAG
TLONGI_14376   AATGCCTCTGCCGATTCACACACCCAAGGGAATAGCTTCTGGAATGCCTTCCAGGC---CGT--C--CAT-T-TCCCATCCACACCCGTC---CGCAGCTCCGTACAATGCAGGCGATCCCTCGAAGATGCGACTGGCAAAGGCCTATAG
TR_60282       CATGCCCCTGCCTATTCACACGCCCAAAGGGATCGTTGCCGGAATGCCTTCCAGGC---CAT--C--CAT-T-TCCCATCCACACCCGTC---CGCAGCCCTTACAACGCAGGCGATCCCTCCAAGATGCGGCTGGCAAAGGCCTATAG
TRICITRO_110   CATGCCCCTGCCTATTCACACACCCAGGGGAATAGTTGCCGGCATGCCTTCGAGGC---CAT--C--CAT-C-TCCCATCCACA-TC--CGTCTGCGGCCCCATACAACGCAGGTGATCCCTCCAAGATGCGGCTGGCAAAGGCTTACAG
THZ_79686      CATGCCTTTGCCTATCCATACCCCAAAGGGCATGCTCGCCGGAATGCCTTCGAGGC---CAT--C--CAT-C-TCTTATCAGCACCCGCCCTCTGCAGCTCCATACAACGCAAGTGACCCTTCCAAGATGCGGCTAGCAAAGGCCTATAG
TH_701679      CATGCCTTTGCCTATCCATACCCCAAAGGGCATGCTCGCCGGAATGCCTTCGAGGC---CAT--C--CAT-C-TCTTATCAGCACCCGCCCTCTGCAGCTCCATACAACGCAAGTGACCCTTCCAAGATGCGGCTAGCAAAGGCCTATAG
A0A1T3C8Z2     CATGCCTTTGCCTATCCATACCCCAAAAGGCATGGTCGCCGGAATGCCTCCGAGAC---CAT--C--CAT-C-TCGCACCAGCACCCGCCCTCTGCAGCTCCATACAACGCAAGCGACCCGTCCAAGATGCGGCTAGCAAAGGCCTATAG
TGAM_8287      CGTTCCCCTGCCCATCCACACGCCAAGGGGCATGGTCGCCGGCATGCCTTCGCGAT---CAT--C--CAT-G-TCCCATCCGTATCCTCCATCTACAGCGCCATACAACGCGAGTGATCCGTCCAAGATGCGCCTCGCCAAGGCCTACAG
OPB37586       CATGCCTTTGCCTATCCATACCCCAAAAGGCATGGTCGCCGGAATGCCTCCGAGAC---CAT--C--CAT-C-TCGCACCAGCACCCGCCCTCTGCAGCTCCATACAACGCAAGCGACCCGTCCAAGATGCGGCTAGCAAAGGCCTATAG
cDNA OPB3758   CATGCCTTTGCCTATCCATACCCCAAAAGGCATGGTCGCCGGAATGCCTCCGAGAC---CAT--C--CAT-C-TCGCACCAGCACCCGCCCTCTGCAGCTCCATACAACGCAAGCGACCCGTCCAAGATGCGGCTAGCAAAGGCCTATAG
cDNA Xlr2*     AATACCTCTCCCTATTCACACCCCAAAAGGCATTGTTGCTGGAATGCCTTCAAGGC---CCT--C--CAT-C-TCTCACCCGCACCCATCATCTGCAGCGCCATACAATGCGGGTGATCCTTCCAAGATGCGGCTCGCAAAAGCCTATAG

               2430      2440      2450      2460      2470      2480      2490      2500      2510      2520      2530      2540      2550      2560      2570
                  *         *         *         *         *         *         *         *         *         *         *         *         *         *         *
Xlr2           TACGCACATTCTACACGTGCTACATGTGCTTCTACACGGCAAATGGGATGCCATATCCATGTTGGACGACGGCGACGACTGGATTACGTCCAAGAGATTCAACGAGTGCGCGTCACATGCCATTTCCGCCTCACAATCGGTCTCGACCAT
Xlr2-000202    TACGCACATTCTACACGTGCTACATGTGCTTCTACACGGCAAATGGGATGCCATATCCATGTTGGACGACGGCGACGACTGGATTACGTCCAAGAGATTCAACGAGTGCGCGTCACACGCCATTTCCGCCTCACAATCGGTCTCGACCAT
TAS_375582     CACGCATATCCTGCATGTGCTGCATGTACTCCTACACGGCAAATGGGATGCCATATCCATGTTGGACGATGGCGACGATTGGATCACGTCCAAGAGATTCAACGAATGCGCATCACATGCCATTTCTGCTTCGCAGTCTGTCTCGACCAT
TA_229644      CACGCACATTCTCCATGTGCTGCATGTGCTCCTCCACGGCAAATGGGATGCCATATCCATGCTGGACGACGGCGATGACTGGATCACATCTAAAAGATTCAACGAGTGCGCGTCACACGCCATTTCTGCGTCGCAGTCCGTTTCGACAAT
TLONGI_14376   CACGCACATCCTGCACGTGTTGCATGTACTGCTGCACGGAAAATGGGATGCCATCTCCATGCTGGACGACGGCGACGATTGGATCACTTCCAAGAGGTTCAACGAGTGCGCGTCACATGCCATCTCTGCGTCGCAGTCTGTCTCGACTAT
TR_60282       CACGCACATCTTGCACGTGCTGCACGTGCTGCTCCATGGAAAATGGGATGCCATCTCCATGCTGGACGACGGCGACGATTGGATCACGTCCAAAAGGTTCAACGAATGCGCGTCGCATGCCATTTCTGCGTCGCAGTCCGTCTCGACTAT
TRICITRO_110   CACGCATATTCTGCACGTGCTGCATGTCCTGCTACACGGCAAGTGGGATGCCATCTCCATGCTGGACGACGGCGACGATTGGATCACGTCCAAGAGGTTTAATGAGTGCGCGTCGCATGCCATTTCTGCGTCACAATCCGTCTCGACCAT
THZ_79686      CACCCACATTCTACACGTATTGCATGTGCTTCTGCACGGCAAATGGGATGCTATATCTATGTTGGACGACGGCGACGATTGGATCACGTCCAAGAGATTCAACGAGTGCGCGTCTCACGCCATTTCTGCGTCGCAATCGGTCTCGACCAT
TH_701679      CACCCACATTCTACACGTATTGCATGTGCTTCTGCACGGCAAATGGGATGCTATATCTATGTTGGACGACGGCGACGATTGGATCACGTCCAAGAGATTCAACGAGTGCGCGTCTCACGCCATTTCTGCGTCGCAATCGGTCTCGACCAT
A0A1T3C8Z2     CGCTCACATTCTGCACGTATTGCATGTGCTTCTACATGGCAAATGGGATGCTATATCTATGTTGGACGACGGCGACGATTGGATCACGTCCAAGAGATTCAACGAGTGCGCGTCTCACGCCATTTCTGCATCACAATCCGTCTCGACCAT
TGAM_8287      CACGCATATTCTCCATGTGCTGCATGTACTCCTGCACGGTAAATGGGATGCCATATCCATGCTGGACGATGGCGATGACTGGATCACATCCAAGAGATTCAACGAGTGCGCGTCACATGCCATTTCTGCGTCGCAGTCTGTTTCAACAAT
OPB37586       CGCTCACATTCTGCACGTATTGCATGTGCTTCTACATGGCAAATGGGATGCTATATCTATGTTGGACGACGGCGACGATTGGATCACGTCCAAGAGATTCAACGAGTGCGCGTCTCACGCCATTTCTGCATCACAATCCGTCTCGACCAT
cDNA OPB3758   CGCTCACATTCTGCACGTATTGCATGTGCTTCTACATGGCAAATGGGATGCTATATCTATGTTGGACGACGGCGACGATTGGATCACGTCCAAGAGATTCAACGAGTGCGCGTCTCACGCCATTTCTGCATCACAATCCGTCTCGACCAT
cDNA Xlr2*     TACGCACATTCTACACGTGCTACATGTGCTTCTACACGGCAAATGGGATGCCATATCCATGTTGGACGACGGCGACGACTGGATTACGTCCAAGAGATTCAACGAGTGCGCGTCACATGCCATTTCCGCCTCACAATCGGTCTCGACCAT
```

```
                   2580      2590      2600      2610      2620      2630      2640      2650      2660      2670      2680      2690      2700      2710      2720
                      *         *         *         *         *         *         *         *         *         *         *         *         *         *         *
Xlr2            CTTGACCATTGATCCAGAGCTTACCTTCATGTCCTATCTTTTTGGTATATATCTACTCCAGGGAAGCTTTATCCTTCTCCTTTTCGCAGATAGGATGCCGCAATTGGGTCCCAACGAGTCGGTGGCACAGGCGTGCGAGAATATTATTCG
Xlr2-000202     CTTGACCATTGATCCAGAGCTTACCTTCATGTCCTATCTTTTTGGTATATATCTACTCCAGGGAAGCTTTATCCTTCTCCTTTTCGCAGATAGGATGCCGCAATTGGGTCCCAACGAGTCGGTGGCACAGGCGTGCGAGAATATTATTCG
TAS_375582      CTTGACGATTGATCCAGAGCTCACCTTCATGTCCTATCTGTTTGGCATATATCTCCTGCAGGGAAGCTTTATCCTTCTTCTCTTTGCGGATAGGATGCCGCAGCTGGGCCCGAACGAATCAGTGGCGCAGGCATGCGAGAATATCATCCG
TA_229644       CTTAACAATCGACCCGGAGCTTACTTTTATGTCCTATCTGTTCGGCATATATCTTCTTCAGGGAAGCTTCATCCTTCTTCTCTTTGCAGACAGGATGCCTCAGCTAGGTCCGAACGAGTCGGTCGCGCAGGCATGCGAGAACATCATTCG
TLONGI_14376    CCTGACCATCGACCCGGAACTGACATTCATGTCCTATCTGTTTGGGATTTATCTGCTGCAGGGGAGTTTCATTCTGTTGCTCTTTGCGGACAGGATGCCGCAGCTTGGTCCCAATGAGTCGGTTGCGCAGGCGTGCGAGAATATCATCCG
TR_60282        CTTGACTATCGATCCGGAACTGACCTTCATGTCCTATCTCTTCGGGATCTATCTGCTGCAGGGGAGTTTCATTCTTTTGCTCTTCGCGGACAGGATGCCGCAGCTTGGTCCCAACGAGTCGGTTGCGCAGGCGTGTGAGACCATTATTCG
TRICITRO_110    TTTGACTATTGATCCCGAACTTACGTTCATGTCCTACCTCTTTGGGATATATCTCCTGCAGGGAAGTTTCATTCTACTGCTCTTCGCGGACAGGATGCCGCAACTTGGCCCCAATGAGTCGGTTGCGCAAGCGTGCGAGAATATCATTCG
THZ_79686       CTTGACCATTGATCCAGAACTTACCTTCATGTCCTACCTTTTTGGCATATATCTGCTTCAAGGAAGCTTCATCCTTCTCCTATTCGCAGACAGGATGCCGCAATTGGGTCCCAACGAGTCGGTAGCGCAGGCCTGCGAGAACATCATTCG
TH_701679       CTTGACCATTGATCCAGAACTTACCTTCATGTCCTACCTTTTTGGCATATATCTGCTTCAAGGAAGCTTCATCCTTCTCCTATTCGCAGACAGGATGCCGCAATTGGGTCCCAACGAGTCGGTAGCGCAGGCCTGCGAGAACATCATTCG
A0A1T3C8Z2      CTTGACCATTGACCCAGAACTTACCTTCATGTCCTATCTTTTTGGCATATATCTGCTTCAAGGAAGCTTCATCCTTCTCCTATTCGCAGACAGGATGCCGCAATTGGGTCCCAACGAGTCGGTGGCACAGGCCTGCGAGAACATCATTCG
TGAM_8287       TTTGACAATCGACCCGGAGCTTACCTTTATGTCTTATCTGTTTGGCATATATCTCCTGCAGGGAAGCTTTATCCTTCTTCTCTTTGCGGACAGGATGCCTCAGCTGGGGCCGAACGAGTCGGTGGCGCAGGCATGCGAGAATATCATTCG
OPB37586        CTTGACCATTGACCCAGAACTTACCTTCATGTCCTATCTTTTTGGCATATATCTGCTTCAAGGAAGCTTCATCCTTCTCCTATTCGCAGACAGGATGCCGCAATTGGGTCCCAACGAGTCGGTGGCACAGGCCTGCGAGAACATCATTCG
cDNA OPB3758    CTTGACCATTGACCCAGAACTTACCTTCATGTCCTATCTTTTTGGCATATATCTGCTTCAAGGAAGCTTCATCCTTCTCCTATTCGCAGACAGGATGCCGCAATTGGGTCCCAACGAGTCGGTGGCACAGGCCTGCGAGAACATCATTCG
cDNA Xlr2*      CTTGACCATTGATCCAGAGCTTACCTTCATGTCCTATCTTTTTGGTATATATCTACTCCAGGGAAGCTTTATCCTTCTCCTTTTCGCAGATAGGATGCCGCAATTGGGTCCCAACGAGTCGGTGGCACAGGCGTGCGAGAATATTATTCG

                   2730      2740      2750      2760      2770      2780                    2790           2800               2810        2820                 2830
                      *         *         *         *         *          *                     *               *                  *           *                    *
Xlr2            AGCCCACGAGGTGTGCGTTGTGACGCTTAGCACAGAGTTTCAGGTATGGAGAGCTC-TACTAAATT-----------TGCCATC-AC---C-CAGACAATAA-T------GCTAACA-TCCAAA---------TAGAAAAA-TTTCCGAA
Xlr2-000202     AGCCCACGAGGTGTGCGTTGTGACGCTTAGCACAGAGTTTCAGGTATGGAGAGCTC-TACTAAATT-----------TGCCATC-AC---C-CAGACAATAA-T------GCTAACA-TCCAAA---------TAGAAAAA-TTTCCGAA
TAS_375582      AGCGCACGAAGTATGCGTTGTAACGTTGAGCACGGAATTTCAGGTA------CCTA-TA-TACATT-----------CACACTC-TT---ATCAGAGAAAGC-GGAAAAAGCTAACATTTGGTG---------GAGAAAAA-CTTCCGAA
TA_229644       CGCGCACGAGGTATGCGTTGTAACGCTGAGCACGGAATTTCAGGTA--CAAAAGTC-CCCCCATTT-----------ATGCATT-GGATTC-TTGTCATGAA-G------TGGAAAA-GTTAATTTTTGTTATTAGAAAAA-CTTTCGAA
TLONGI_14376    AGCTCATGAGGTGTGTGTTGTGACGCTGAGTACGGAGTTCCAGGTATGCAGTCCTC-TTCCCTTGT-----------GTACCTT-TT---T-CTGAG----A-T------GCTAACA-CTCGAG---------CAGAAAAACTTTC-GTA
TR_60282        AGCTCACGAGGTGTGCGTTGTGACGCTGAGCACAGAATTCCAGGTATGCAGCCCTCGTCCCTTTTA-----------TGTGAGA-AA---T-GCTGC-AGGA-T------GTTGACA-TTTAGC---------TAGAAAAA-CTTTCGTA
TRICITRO_110    AGCTCACGAGGTGTGCGTTGTGACGCTGAGTACAGAGTTTCAGGTATG--CAGCCCCTACTCTTGT-----------GCAAATT-AC---C-TGCAAGATAGGT------ATTGACG-CTCAGA---------CAGAAAAA-CTTTCGTA
THZ_79686       AGCCCATGAGGTGTGCGTTGTAACGCTGAGTACAGAGTTTCAGGTATGGAGAGCAC-T-CATAACT-----------TCCACCC-AC---C-CAGA-AATAA-T------GCTAACT-TCCAAA---------CAGAAAAA-CTTCCGTA
TH_701679       AGCCCATGAGGTGTGCGTTGTAACGCTGAGTACAGAGTTTCAGGTATGGAGAGCAC-T-CATAACT-----------TCCACCC-AC---C-CAGA-AATAA-T------GCTAACT-TCCAAA---------CAGAAAAA-CTTCCGTA
A0A1T3C8Z2      AGCTCATGAGGTGTGCGTTGTGACGTTGAGTACAGAGTTTCAGGTATGCAGAGCAC-T-CATAATT-------------CCAGCTAC---C-CAGA-AACAA-T------GCTAACT-TCCACA---------TAGAAAAA-CTTCCGTA
TGAM_8287       AGCGCATGAGGTATGCGTTGTAACGCTGAGCACGGAATTCCAGGTATG-AAGTCCC-TCATTGATTGAGTGTTTTATTGTCATG-AA---G-TGGA----AA-T------GTTAACA-T-GATG---------TAGAAAAA-CTTTCGAA
OPB37586        AGCTCATGAGGTGTGCGTTGTGACGTTGAGTACAGAGTTTCAGGTATGCAGAGCAC-T-CATAATT-------------CCAGCTAC---C-CAGA-AACAA-T------GCTAACT-TCCACA---------TAGAAAAA-CTTCCGTA
cDNA OPB3758    AGCTCATGAGGTGTGCGTTGTGACGTTGAGTACAGAGTTTCAG---------------------------------------------------------------------------------------------AAAAA-CTTCCGTA
cDNA Xlr2*      AGCCCACGAGGTGTGCGTTGTGACGCTTAGCACAGAGTTTCAG---------------------------------------------------------------------------------------------AAAAA-TTTCCGAA
                                                           Intron 5

                2840      2850      2860      2870      2880      2890      2900      2910      2920      2930      2940      2950      2960
                   *         *         *         *         *         *         *         *         *         *         *         *         *
Xlr2            CAGTTCTACGATCTACTCTATACAGCGTTCAAGGCGCAGGAACTACGAATTGGGATGAGCATCGTTCACGACGAAGGGCTCTTTCTTTGTACCGGTGGACAAAGGGGGCCAAGGGGCTGGCACTGTAA
Xlr2-000202     CAGTTCTACGATCTACTCTATACAGCGTTCAAGGCGCAGGAACTACGAATTGGGATGAGCATCGTTCACGACGAAGGGCTCTTTCTTTGTACCGGTGGACAAAGGGGGCCAAGGGGCTGGCACTGTAA
TAS_375582      CAGTATTGCGGTCAACTCTGTATAGCGTTCAAGGCGCAGGGACGACGAATTGGGATGAGCATCGCTCACGACGAAGAGCTTTATCTTTATATAGGTGGACAAAGGGAGCTAGGGGGTTGGCATTATGA
TA_229644       CAGTGTTACGGTCAACTCTCTATAGCGTCCAGGGCGCGGGAACGACGAATTGGGATGAACATCGTTCAAGACGAAGAGCTCTGTCTTTGTACAGATGGACGAAAGGAGCCAGGGGACTGGCATTATGA
TLONGI_14376    CCGTTTTGCGATCTACGCTTTACAGTGTTCAAGGCGCAGGAACTACAAATTGGGACGAGCACCGTTCACGACGCCGGGCTTTGTCGCTGTATAGATGGACAAAGGGGGCCAAGGGACTGGCATTGTGA
TR_60282        CCGTTTTGCGATCTACGCTTTATAGTGTTCAAGGTGCAGGAACTACGAATTGGGACGAGCATCGTTCACGACGCAGAGCTTTGTCGCTGTATAGATGGACAAAGGGGGCCAAGGGACTGGCATTGTGA
TRICITRO_110    CGGTCTTGCGATCTACGCTATATAGTGTTCAAGGCGCTGGAACTACCAACTGGGACGAGCATCGCTCACGACGGAGAGCTTTGTCGTTGTATAGATGGACAAAGGGGGCCAAGGGACTGGCATTGTAA
THZ_79686       CGGTCCTACGATCCACTCTATACAGCGTTCAGGGCGCAGGAACCACGAATTGGGATGAGCATCGCTCACGACGCAGAGCTCTGTCCTTGTACAGGTGGACAAAGGGAGCCAAGGGGCTGGCATTGTAG
TH_701679       CGGTCCTACGATCCACTCTATACAGCGTTCAGGGCGCAGGAACCACGAATTGGGATGAGCATCGCTCACGACGCAGAGCTCTGTCCTTGTACAGGTGGACAAAGGGAGCCAAGGGGCTGGCATTGTAG
A0A1T3C8Z2      CGGTCCTACGATCCACTCTATACAGCGTTCAGGGCGCGGGAACTACGAATTGGGATGAGCATCGCTCACGACGCAGAGCTCTATCCTTGTACAGGTGGACAAAGGGAGCCAAGGGGCTGGCATTGTAG
TGAM_8287       CAGTATTACGATCCACTCTGTATAGCGTCCAGGGAGCGGGAACGACGAACTGGGATGAGCATCGTTCGAGACGAAGAGCTCTGTCTTTGTACAGATGGACGAAAGGAGCCAAGGGTCTAGCATTATGA
OPB37586        CGGTCCTACGATCCACTCTATACAGCGTTCAGGGCGCGGGAACTACGAATTGGGATGAGCATCGCTCACGACGCAGAGCTCTATCCTTGTACAGGTGGACAAAGGGAGCCAAGGGGCTGGCATTGTAG
cDNA OPB3758    CGGTCCTACGATCCACTCTATACAGCGTTCAGGGCGCGGGAACTACGAATTGGGATGAGCATCGCTCACGACGCAGAGCTCTATCCTTGTACAGGTGGACAAAGGGAGCCAAGGGGCTGGCATTGTAG
cDNA Xlr2*      CAGTTCTACGATCTACTCTATACAGCGTTCAAGGCGCAGGAACTACGAATTGGGATGAGCATCGTTCACGACGAAGGGCTCTTTCTTTGTACCGGTGGACAAAGGGGGCCAAGGGGCTGGCACTGTAA
```

Figure S3. Alignment of Xlr2 encoding gene orthologs in diverse Trichoderma species. The alignment includes cDNA obtained from our *Trichoderma virens* Gv29.8 RNA-seq data (cDNA Xlr2*, Castañeda-Casasola, Lawry et al., in preparation), predicted mRNA sequences from *T. guizhouense* (cDNA OPB3758) and *T. parareesei* (OTA07803.1), as well as the extended 5′ region of various *Trichoderma* gene orthologs. The presence of the microexon sequence is observed in all Xlr2 orthologs from the *Trichoderma* species analyzed in this study. Introns are indicated by orange bars. The sequence Xlr2-000202 corresponds to the ortholog in *T. virens* FT-333, the gene reported in NCBI corresponds to TrVFT333_000202. It was obtained from a search using NCBI Blast. The orthologous gene was identified on chromosome 1 (https://www.ncbi.nlm.nih.gov/nuccore/CP071115.1?from=816716&to=819677). The correspondence names between this figure and figure 4B is as follow:

**This Figure**	**Figure 4B**
Xlr2	PP712105
Xlr2-000202	Xlr2-000202**
TAS_375582	TAS_375582
TA_229644	Triat2_229644
TLONGI_14376	Trilo3_1437635
TR_60282	TRQM6a_60282
TRICITRO_110	Trici4_1109904
THZ_79686	Triha1_79686
TH_701679	Trihar1_701679
A0A1T3C8Z2	A0A1T3C8Z2
TGAM_8287	Trigam1_8287
OPB37586	OPB37586
cDNA OPB3758	cDNA OPB3758
cDNA Xlr2*	cDNA PP712105

** this is only present in this table and corresponds to TrVFT333_000202 encoding gene see the figure legend.
