# Supplementary material for: Unveiling a Microexon Switch: Novel Regulation of the Activities of Sugar Assimilation and Plant-Cell-Wall-Degrading Xylanases and Cellulases by Xlr2 in Trichoderma virens
Source: Int J Mol Sci. 2024 May 9;25(10):5172. doi: 10.3390/ijms25105172 (PMC11121469; doi:10.3390/ijms25105172)
Supplement: Supplementary file 1 [file ijms-25-05172-s001.zip › Figure S4 new number.pdf]

```
                                            Zn(2)-C6 fungal-type DNA-binding domain

                      10         20         30         40         50         60         70         80         90        100        110        120        130                140
                       *          *          *          *          *          *          *          *          *          *          *          *          *                  *
Xlr2          MAAEADGQERDRPEPLVASLMQRSETPDTEHARPRKRTRRACDKCSASRTRCDGECPCRRCEDYGYTCRYNREVKKRGRLPASATANNNHNTNGSINSRRDSWPLSHRRNSI-RNDSNLVRSGNGNGSSNS-------NSNGISQASCSS
OTA07803.1    MAAEADGQERDRPEPLVGSLAQHSETPDTEHARPRKRTRRACDKCSASRTRCDGECPCRRCEDYGYTCRYNREVKKRGRLPASATANN----TGSISARRESWPLSTRRGSI-RNDSPLSRNANGNSNGSSSNGHAMGYGNAISQASPSS
OPB37586      MAAEADGQERDRPEPLVASLMHRSETPDTEHARPRKRTRRACDKCSASRTRCDGECPCRRCEDYGYTCRYNREVKKRGRLPASATANNHNTNGSSINARRDSWPQSHRRGSI-RNDSNLGRSSNGNSNSNS-------NSNGISQASCSS
TH_79686      MAAEADGQERDRPEPLVASLMHRSETPDTEHARPRKRTRRACDKCSASRTRCDGECPCRRCEDYGYTCRYNREVKKRGRLPASATANNHNTNGSSINARRDSWPQSHRRGSI-RNDSILGRSSNGNSNSNS-------NSNGISQASCSS
TAS_375582    MATEADGEQRDRPQPLVASGHDRGSTPDAEHARPRKRTRRACDKCSASRTRCDGEYPCRRCEDYGYTCRYNREVKKRGRLPASATANNHNNSNGSISNRRDSWASSARRGSIIRRDSNIGHSGNGNG-----------NGNGISRASVSS
TH_701679     MAAEADGQERDRPEPLVASLMHRSETPDTEHARPRKRTRRACDKCSASRTRCDGECPCRRCEDYGYTCRYNREVKKRGRLPASATANNHNTNGSSINARRDSWPQSHRRGSI-RNDSILGRSSNGNSNSNS-------NSNGISQASCSS
TLONGI_14376  MAAEADGQERHRPEPLVAPLAQRSETPDTEHARPRKRTRRACDKCSASRTRCDGECPCRRCEDYGYTCRYNREVKKRGRLPASATANN-----GNISARRDSWPLSTRRGSI-RNDSHPSRNANGNINSSNSNGHAMANGIAISQTSSSS
TR_60282      MAAEADGQERDRPEPLVGSLAQHSETPDTEHARPRKRTRRACDKCSASRTRCDGECPCRRCEDYGYTCRYNREVKKRGRLPASATANN-----GNISARRESWPLSTRRGSI-RNDSPLSRNANGNSNGSSSNGHAMGYGNAISQASPSS
TRICITRON_11  MAAEADGQERHRRGSLVASVTQRSETPDTEHARPRKRTRRACDKCSASRTRCDGECPCRRCEDYGYTCRYNREVKKRGRLPASATGNN----HSNISARRDSWPLSSRRGSI-RNDPHLDRNANGNSNSNSSNGHAMGNGSVISQASPSP
TGAM_8287     MATEADGEQRDRPQPLVASEHDRGSTPDAEHARPRKRTRRACDKCSASRTRCDGEYPCRRCEDYGYTCRYNREVKKRGRLPASATAN--HKSNGSISNRRDSWASSTRRGSIIRRESNIGHTSNGSS-----------NGNGISRASVSS
              **:****::*.*  .**..  .:..***:************************* ****************************.*.     ..*. **:**. * **.** *.:.   :..**. .            .  **::* **

                                                                              NLS

                     150        160        170        180        190        200        210        220        230        240        250        260        270        280        290
                       *          *          *          *          *          *          *          *          *          *          *          *          *          *          *
Xlr2          PSATSASISSSTVGQHPFADPVDAASAGPDGVTLD-RDQVTMSDTRQDVLHAISQPYSQSRTAPPLSVPSLIQVGLQPPPPQRSAIGNPLSVNVFDGQFADSEVVLSDESAGYPSPVNGRPHIEAGNTRQHRGSFKSTPSHERNRESFST
OTA07803.1    LSATSPSISSSTVAQHPFDDPTDATSARPEGAPLDPRDRAAMSDVRPDVLHSISAPFPQSRPAPPLSVPSLSQVGLQPPSAQRASISNPLSVNVFDGQFGDSEVVVSDESVGYPSPINTRPHAEL--NRQHRASGKSTHSHERNRESFST
OPB37586      PSA---SISSSTVGQHPFADTVDATSAGPESVTLDQRDQVTVPDARQDMLHSISQPYSSGRTAPPLSVPSLIQVGLQPPPPQRSSIGNSLSVSVFDGQFADSEVVLSDESAGYPSPVNGRAPIEAGSARQHRGSFKSTPSHERNRESFST
TH_79686      PSA---SISSSTVGQHPFADPVDATSAGPESVTLDQRDQVTVPDARQDMLHSISQPYSQSRTAPPLSVPSLIQVGLQPPPPQRSSIGNSLSVSVFDGQFADSEVVLSDESAGYPSPVNGRPPIETGSARQHRGSFKSTPSHERNRESFST
TAS_375582    PSATSASISSSTAARRSLTDPVDVTTATTDD------DQIALQDSRQDVLHSISQPYPQSRPAPPLSVPSLIQVGIQPPMPPRSSISNSLSVNVFEGQFGDSEVALSDGSVGYPSPVDRRPHVEAANSRQHRGSFKSTPSIERNRESFST
TH_701679     PSA---SISSSTVGQHPFADPVDATSAGPESVTLDQRDQVTVPDARQDMLHSISQPYSQSRTAPPLSVPSLIQVGLQPPPPQRSSIGNSLSVSVFDGQFADSEVVLSDESAGYPSPVNGRPPIETGSARQHRGSFKSTPSHERNRESFST
TLONGI_14376  PSATSPSISSSTVAQHPFGDPTDATSARPEGVPLDPRDRVTMPDARQDVLHSISAPFPQSRPAPPLSVPSLSQVGLQPPPAQRASISHPLSVNVFDGQFGDSEVVLSDESVGYPSPVNTRPHAEV--NRQHRASFKSTHSHERNRESFST
TR_60282      LSATSPSISSSTVAQHPFDDPTDATSARPEGAPLDPRDRVAMSDVRPDVLHSISAPFPQSRPAPPLSVPSLSQVGLQPPSAQRASISNPLSVNVFEGQFGDSEVVVSDESVGYPSPINTRPHAEL--NRQHRASGKSTHSHERNRESFST
TRICITRON_11  -SATSPSISSSTVAQHPFAGPADATSARPEGVPLDPRDRVTMSDARQDVLHSISAPFPQSRPAPPLSVPSLTQVGLQPPPAQRASISNPLSVNVFDGQYGDSEVILSDESAGYPSPVNTRPHAEV--NRQHRASFKSTHSHERNRESFST
TGAM_8287     PSA---SISSSSAARHSLTDAVDVTTATTED------DQITLQDARQDVLHSISQPYPQSRPAPPLSVPSLIQVGIQPPMPPRSSISNPLSVNVFDGQFGDNEVALSDGSVGYPSPVDRRSHAEAANARQHRGSFKSTPSHERNRESFST
                    * .***:..::.: ...*.::* .:      *: :: * * *:**:** *:...*.********* ***:***  . *::*.:.***.**:**:.*.** :** *.*****:: *.  *   ****.* *** * *********

                       300        310        320        330        340        350        360        370        380        390        400        410        420        430        440
                         *          *          *          *          *          *          *          *          *          *          *          *          *          *          *
Xlr2          AAT-AHDSGALMLDFFHKAPNEDCWYKFLEPILPYIRNIIPASVACDLLDIFLTDPGSSLFRVASPYILTRVFRKKSIIHPTAPRYTTPALLATILWCVAQTADVMMLHVPGSRAKVVNDLYDLATSLISERDPDRWRRIHGGLRAENET
OTA07803.1    AATTSHDVGGLMLDFFHRAPNEDCWYKFLEPILPYIRNIIPASVACDLLDIFLTDPGSSLFRVASPYILTRVFRKKSIVHPTNPRYTTPALLATILWCVAQTADVMMLHVPGSRAKVVNDLYDLATSLISERDPDRWRRIHGGLRAENEA
OPB37586      AAT-GHDAAGLMLDFFHKAPNEDCWYKFLEPILPYIRNIIPASVACDLLDIFLTDPGSSLFRVASPYILTRVFRKKSIVHPTNPRYTTPALLATILWCVAQTADVMMLHVPGSRAKVVNDLYDLATSLISERDPDRWRRIHGGLRAENET
TH_79686      AAT-GHDAAGLMLDFFHKAPNEDCWYKFLEPILPYIRNIIPASVACDLLDIFLTDPGSSLFRVASPYILTRVFRKKSIVHPTNPRYTTPALLATILWCVAQTADVMMLHVPGSRAKVVNDLYDLATSLISERDPDRWRRIHGGLRAENET
TAS_375582    N---SHDAGGLMLDFFHKAPNDDCWYKFLEPILPYIRNIIPASVACDLLEIFLTDPGSSLFRVASPYILTRVFRKKSITHPTNPRYTTPALLATILWCVAQTADVMMLHVPGTRAKIVNDLYDLATSLISERDPDRWRRIHGGLRAENET
TH_701679     AAT-GHDAAGLMLDFFHKAPNEDCWYKFLEPILPYIRNIIPASVACDLLDIFLTDPGSSLFRVASPYILTRVFRKKSIVHPTNPRYTTPALLATILWCVAQTADVMMLHVPGSRAKVVNDLYDLATSLISERDPDRWRRIHGGLRAENET
TLONGI_14376  AATTNHDAGGLMLDFFHKAPNEDCWYKFLEPILPYIRNIIPASVACDLLDIFLTDPGSSLFRVASPYILTRVFRKKSIVHPTNPRYTTPALLATILWCVAQTADVMMLHVPGSRAKVVNDLYDLATSLISERDPDRWRRIHGGLRAENEA
TR_60282      AATTSHDVGGLMLDFFHRAPNEDCWYKFLEPILPYIRNIIPASVACDLLDIFLTDPGSSLFRVASPYILTRVFRKKSIVHPTNPRYTTPALLATILWCVAQTADVMMLHVPGSRAKVVNDLYDLATSLISERDPDRWRRIHGGLRAENEA
TRICITRON_11  AATASHDAGGLMSDFFHKAPNEDCWYKFLEPILPYIRNIIPASVACDLLDIFLTDPGSSLFRVASPYILTRVFRKKSIVHPTNPRYTTPALLATILWCVAQTADVMMLHVPGSRAKVVNDLYDLAVSLISERDPDRWRRIHGGLRAENET
TGAM_8287     N---SHDAGGLMLDFFHKAPNDDCWYKFLEPILPYIRNIIPASVACDLLEIFLTDPGSSLFRVASPYILTRVFRKKSITHPTNPRYTTPALLATILWCVAQTADVMMLHVPGTRAKIVNDLYDLATSLISERDPDRWRRIHGGLRAENET
                  **  ..** ****:***:***************************:*************************** *** **************** *************:***:********.***********************:
```

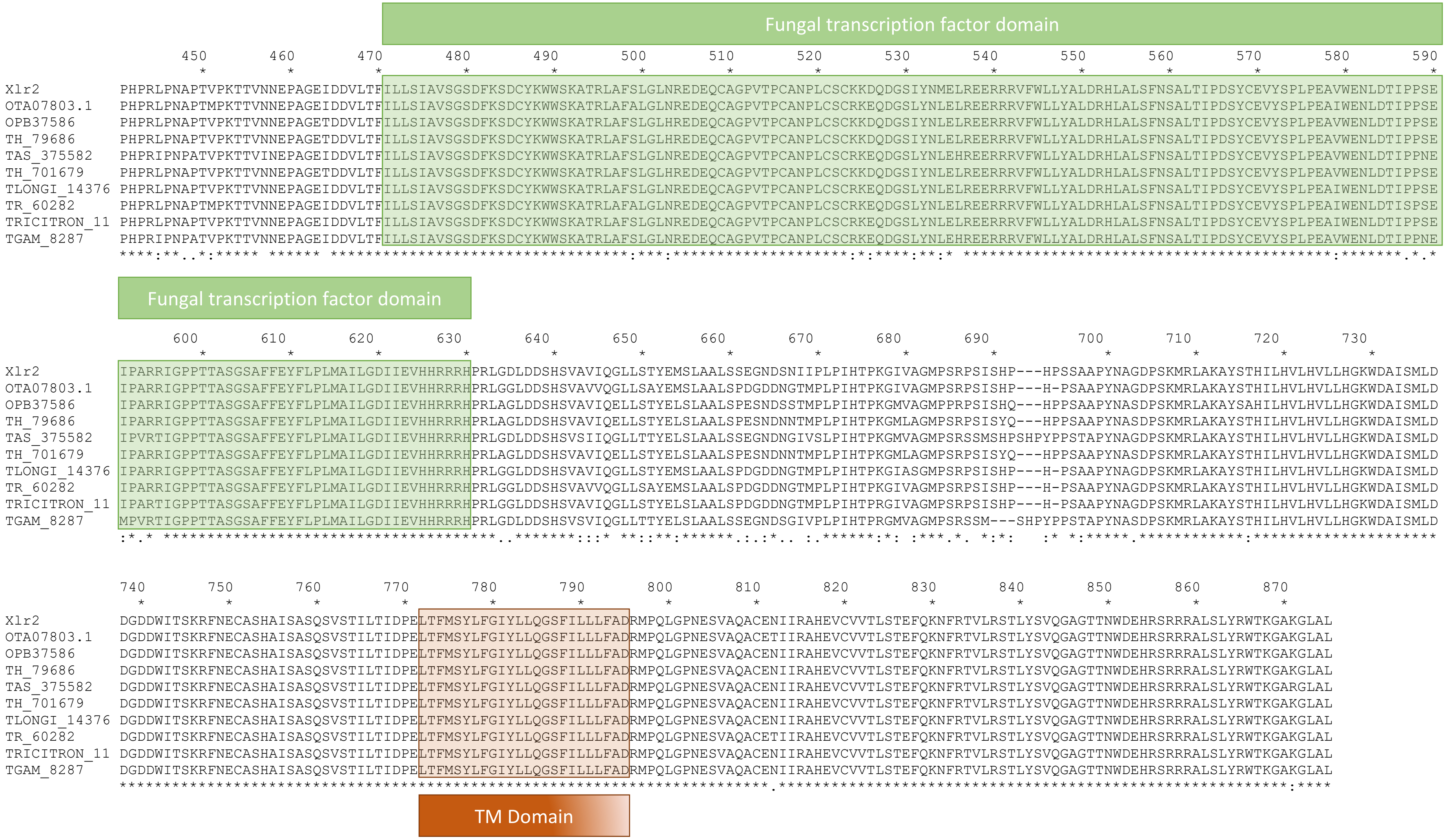

Figure S4: Alignment of Xlr2 Similar Proteins from *Trichoderma*. The alignment was done based on manual annotation. The microexon is highly conserved and encodes the amino acid sequence PCRRCE, which includes two cysteines (C) in the Zn(II)2Cys6 domain. Additional domains identified using InterProScan (https://www.ebi.ac.uk/interpro/search/sequence/) are indicated in different colours.
