# Supplementary material for: Unveiling a Microexon Switch: Novel Regulation of the Activities of Sugar Assimilation and Plant-Cell-Wall-Degrading Xylanases and Cellulases by Xlr2 in Trichoderma virens
Source: Int J Mol Sci. 2024 May 9;25(10):5172. doi: 10.3390/ijms25105172 (PMC11121469; doi:10.3390/ijms25105172)
Supplement: Supplementary file 1 [file ijms-25-05172-s001.zip › Figure S5 new number.pdf]

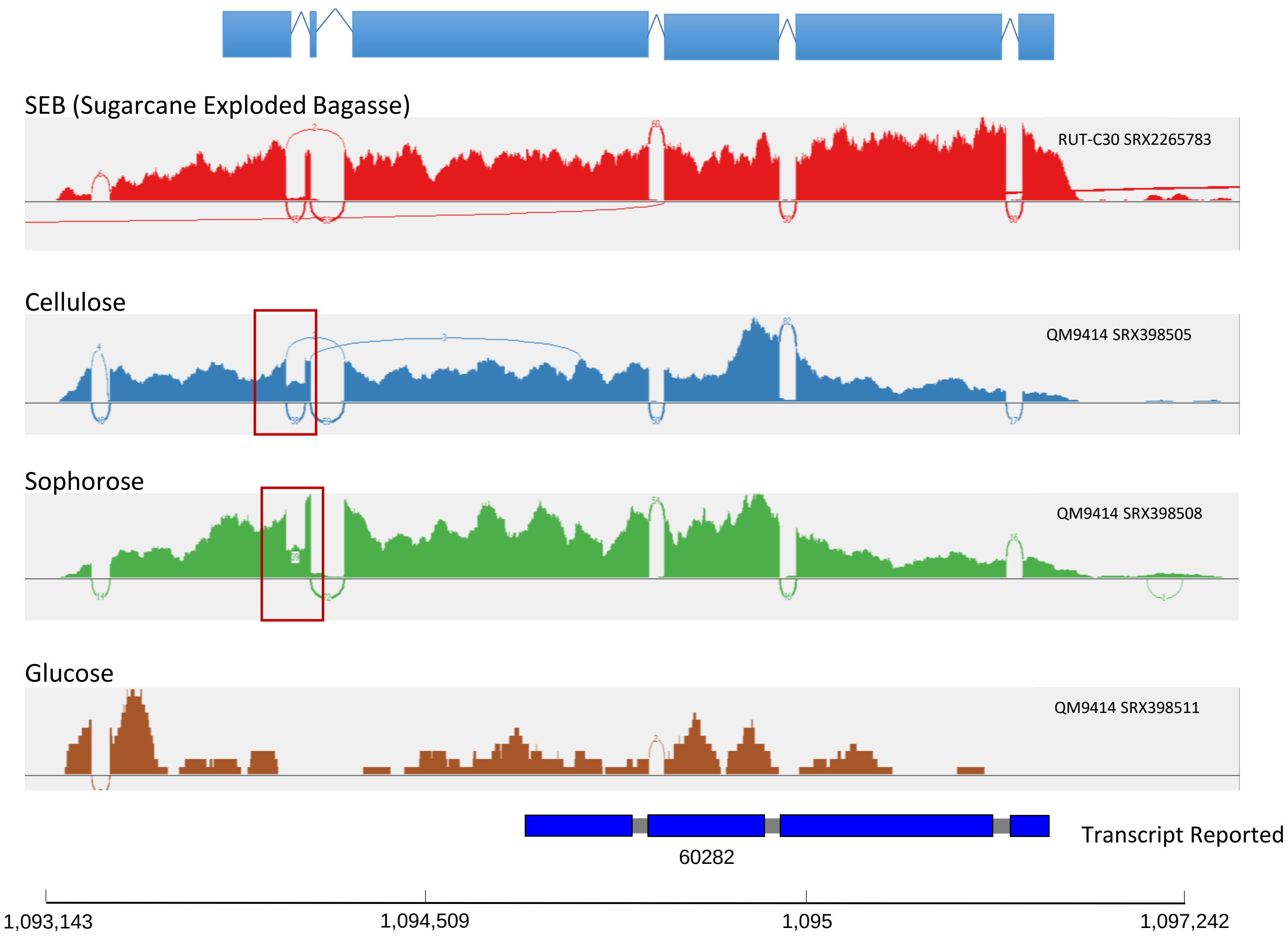

Figure S5. Microexon biology in the ortholog of Xlr2 in *T. reesei*. The partial mapping of publicity available RNA-seq was mapped using Bowtie2 in the Galaxy platform. The mapping of the transcriptome reads from *T. reesei* was visualised using the Integrative Genomics Viewer (IGV) visualization software. SRX2265783: *Trichoderma reesei* RUT-C30 growing on SEB 6h - Replicate 2, SRX398505: GSM1297497: QM9414 Cellulose 1; *Trichoderma reesei*; RNA-Seq, SRX398508: GSM1297500: QM9414 Sophorose 1; *Trichoderma reesei*; RNA-Seq, SRX398511: GSM1297503: QM9414 Glucose 1; *Trichoderma reesei*; RNA-Seq). The first intron not spliced in Cellulose and Sophorose is indicated with a red rectangle. The partial transcript reported in Mycocosm is shown for comparison (https://mycocosm.jgi.doe.gov/cgi-bin/dispGeneModel?db=Trire2&id=60282).
