# Supplementary material for: Unveiling a Microexon Switch: Novel Regulation of the Activities of Sugar Assimilation and Plant-Cell-Wall-Degrading Xylanases and Cellulases by Xlr2 in Trichoderma virens
Source: Int J Mol Sci. 2024 May 9;25(10):5172. doi: 10.3390/ijms25105172 (PMC11121469; doi:10.3390/ijms25105172)
Supplement: Supplementary file 1 [file ijms-25-05172-s001.zip › Figure S6 new number.pdf]

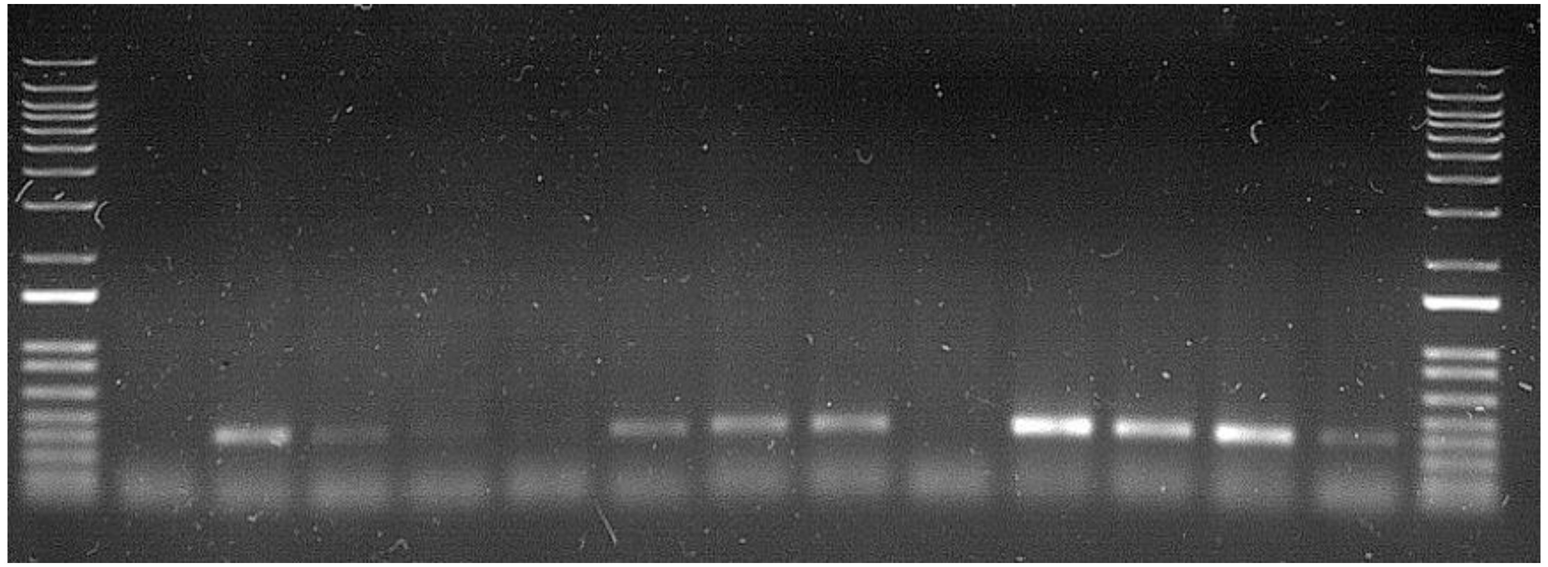

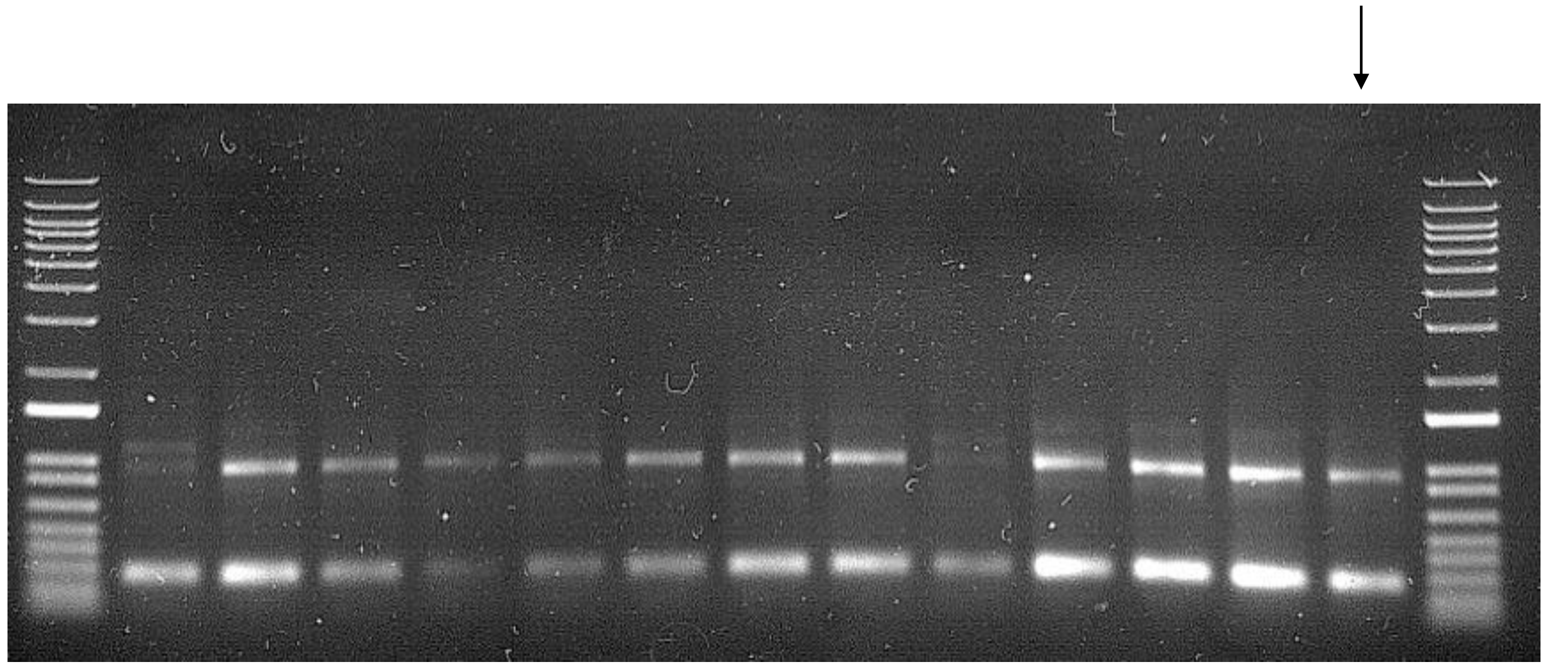

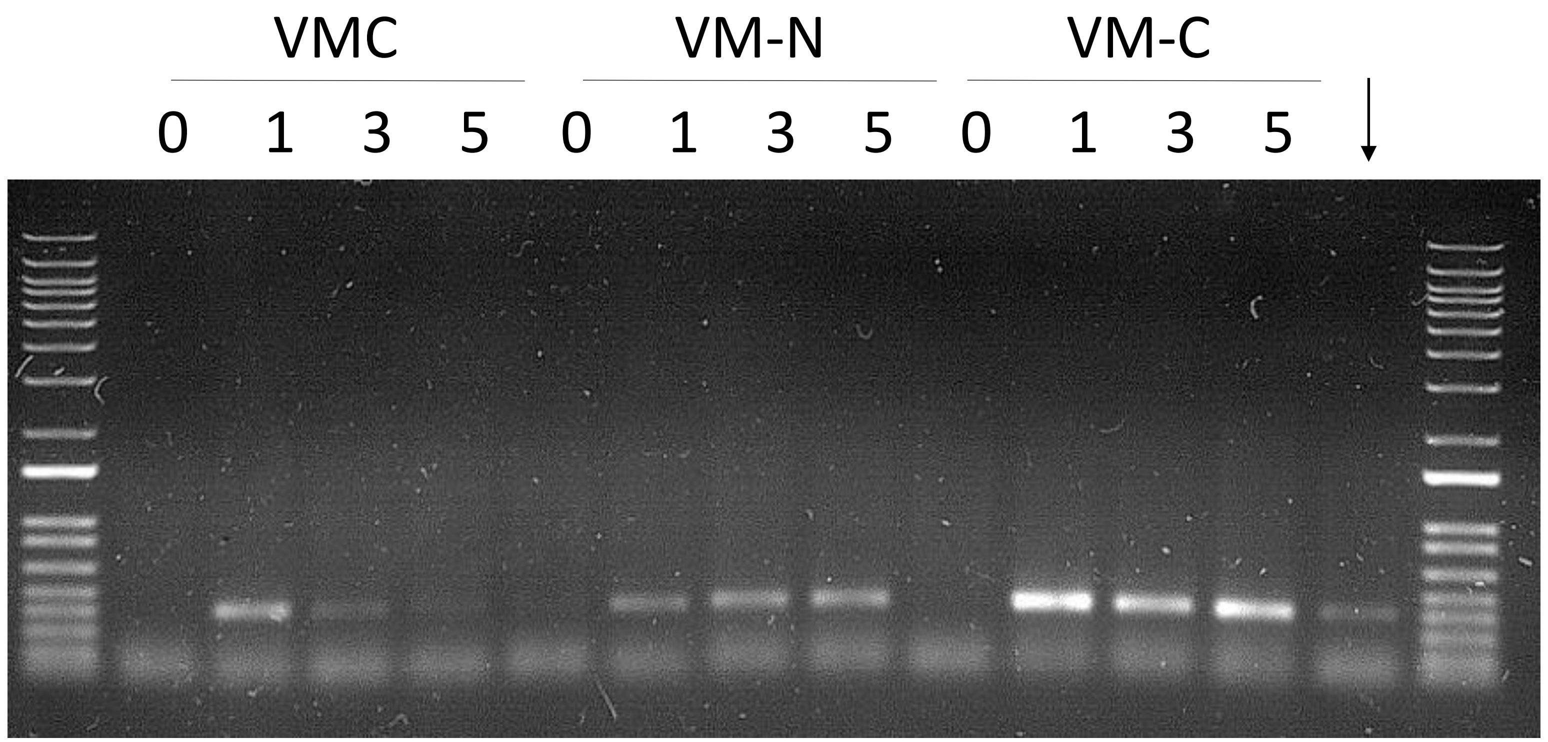

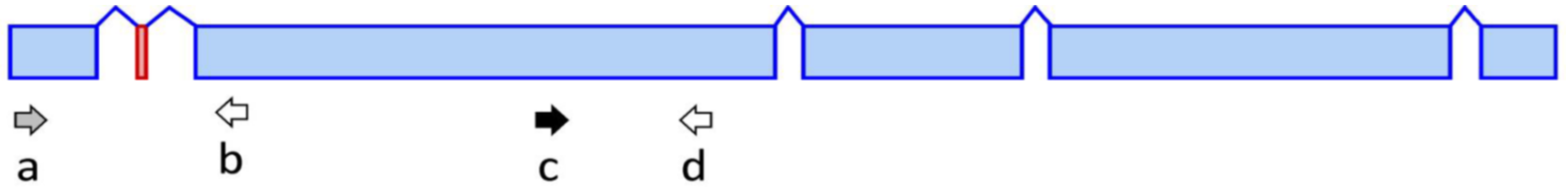

The sample with the arrow was not included in the current study due to correspond
To another condition of *Trichoderma* no discussed in the current manuscript

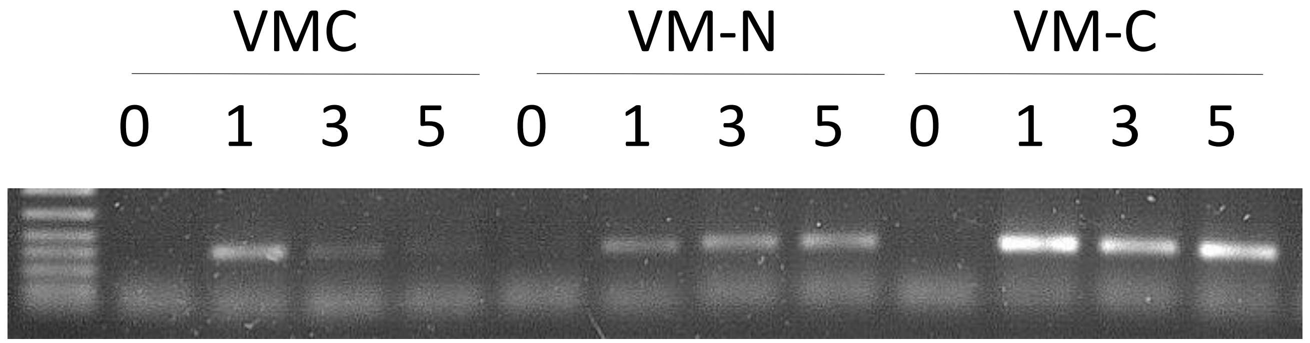

a/b gDNA: 585 bp
a/b cDNA: 414 bp

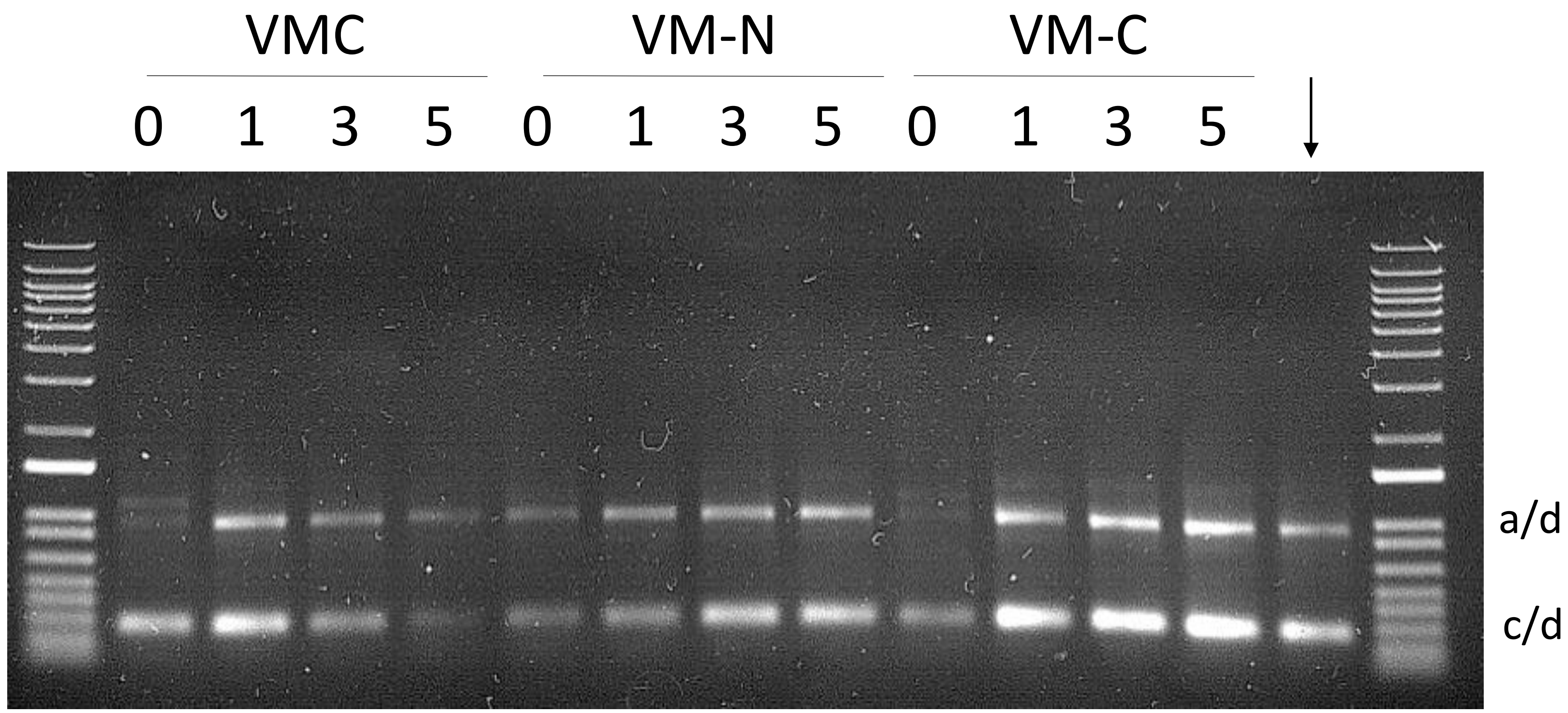

The figure was cut due to we used three primers (a/c/d). To simply the explanation we cut the figure without modifying the interpretation

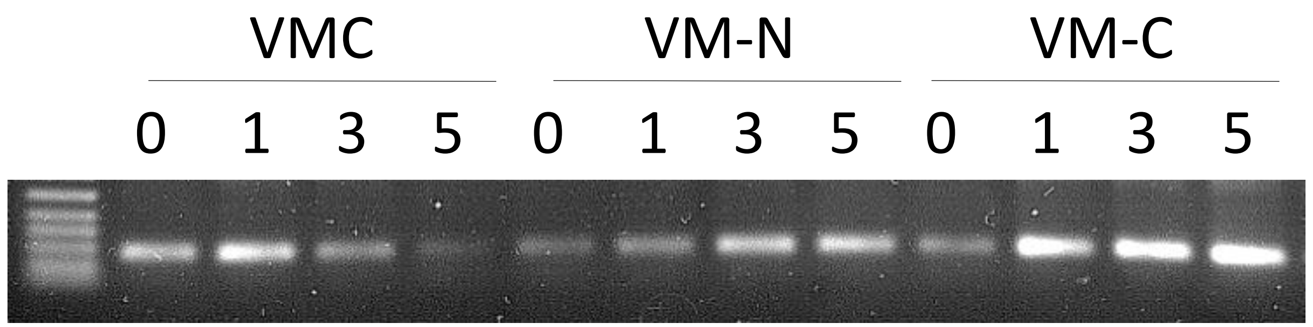

a/d gDNA: 1,110 bp
a/d cDNA: 940 bp

c/d gDNA: 250 bp
c/d cDNA: 250 bp

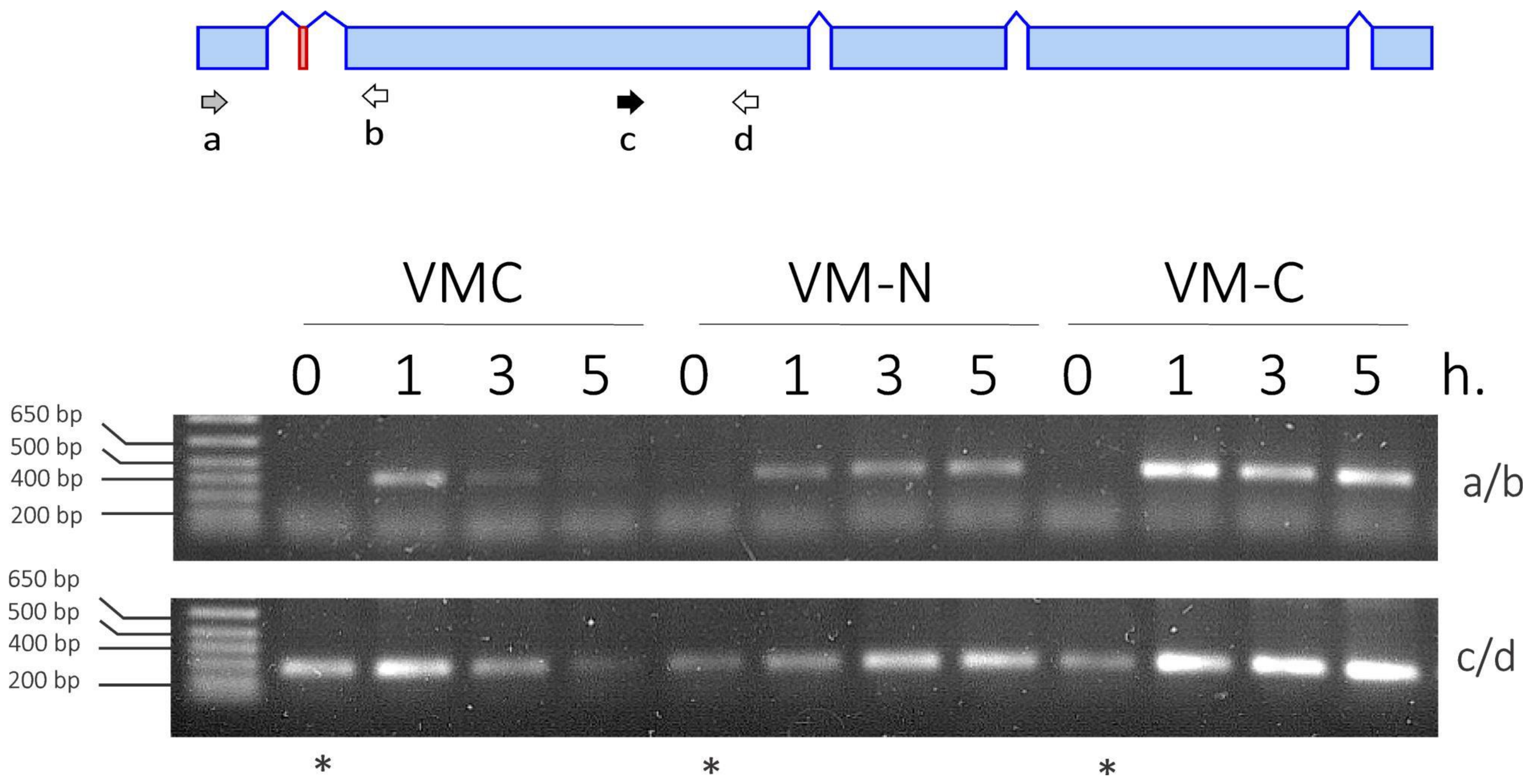

a
b
c
d
VMC
VM-N
VM-C
0 1 3 5 0 1 3 5 0 1 3 5 h.
650 bp
500 bp
400 bp
200 bp
a/b
650 bp
500 bp
400 bp
200 bp
c/d
*
*
*
