# Supplementary material for: Unveiling a Microexon Switch: Novel Regulation of the Activities of Sugar Assimilation and Plant-Cell-Wall-Degrading Xylanases and Cellulases by Xlr2 in Trichoderma virens
Source: Int J Mol Sci. 2024 May 9;25(10):5172. doi: 10.3390/ijms25105172 (PMC11121469; doi:10.3390/ijms25105172)
Supplement: Supplementary file 1 [file ijms-25-05172-s001.zip › Table S1.docx]

**Table S1.** Primers used in this study

| **Primer Name** | **Sequence (5’ -> 3’)** | **Experiment** |
| --- | --- | --- |
| 47927 OPL | GCCGAGTCAGCCACGAGAATAG | Xlr2 Deletion |
| 47927OPR | CCTCGTGAATCCGCCTCTCA | Xlr2 Deletion |
| 47927 LF-L | AAAGGCAAGGCTCATGCCATTTAG | Xlr2 Deletion Left border |
| 47927 LF R SfiI | AACGGGCCATCTAGGCCAACGGGCCTTGTGGAAGAAGTC |  |
| 47927 RF L SfiI | GGCCTGAGTGGCCTTGTACCGGTGGACAAAG | Xlr2 Deletion Right border |
| 47927 RF-R | TTTCAATAGGTCGTCCATCAG |  |
| 47927 LFAcc65I | AAAGGTACCAGCCCTTTGTCGTGTTTC | Xlr2 Deletion subcloning in vector |
| 47927 RF XbaI | TTTTCTAGACAATAGGTCGTCCATCAG |  |
| M13 Forward | TGTAAAACGACGGCCAGT | Sequencing |
| M13 Reverse | CAGGAAACAGCTATGAC | Sequencing PCR |
| oAM-LU347 | GGATGCCTCCGCTCGAAGTA | HY |
| oAM-LU348 | CGTTGCAAGACCTGCCTGAA | YG |
| oAM-LU357 | ttaGCGGCCGCCCGACGTTAACTGATATTGAAGGAGCA | Hygromycin amplification with Not1 restriction sites bordering |
| oAM-LU358 | ttcGCGGCCGCCGTTAACGGAACCCGGTCGG | Hygromycin amplification with Not1 restriction sites bordering |
| AM-LU702 | AAGGTCTCtAATGGCAGCTGAAGCCGACGGTCAA | Amplification of the Xlr2 long (PCR) |
| AM-LU703 | AAGGTCTCtAATGTCTGACACTCGCCAAGATGTG | Amplification Xlr2 short /Expression level Xlr2 (PCR/qPCR) |
| AM-LU704 | TTGGTCTCtAAGCCAGTGCCAGCCCCTTGGCCCCCTTT | C-terminal Xlr2 |
| AM-LU708 | TGGGGCCTTGTGGAAGAAGTC | Amplification ORF /Expression level Xlr2 (qPCR) |
| AM-LU821 | AATGGTACCTTCTGCCATAGGTAATCCTTT | Xlr2 orf |
| oCC7-UPP | AACTTAAAGGAATTGACGGAAG | Expression level 18S (qPCR ) |
| oCC8-UPP | GCATCACAGACCTGTTATTGCCTC |  |
| AM-LU650 | AATGGTACCCCGCATAATAACCAGTCGCC | Amplification microexon (RT-PCR) |
| RL-LU51LR | TAATCTAGACTACCGTTCAAGTTGAATAGAT |  |
| GtR BsaI Fw | GGTCTCt TAAC CTCGACTCTAGAGGATCCTCT | Amplification geneticin resistance gene |
| GtR BsaI Rv | GGTCTCa ACAT CGTCGTCCAGGCGGTGAGCAC |  |
| tef1 BsaI Fw | GGTCTCt ACAA GTACCAGTACATCGTCCGAG | Amplification tef1 Promoter |
| tef1 BsaI Rv | GGTCTCa CATT CTCGACGTGAGTCTTCT |  |
| Ttrp BsaI Fw | GGTCTCt GCTT CTAGTGATTTAATAGCTCC | Amplificationtrp1 Terminator |
| Ttrp BsaI Rv | GGTCTCc GTTA GGTACCTGTGCATTCTGG |  |
